# Integrated ERT, petrophysics and borehole logging for geotechnical zonation and sustainable coastal development in Ras El-Hekma, Egypt

**DOI:** 10.1038/s41598-026-60990-0

**Published:** 2026-07-14

**Authors:** Raghda M. Abd Elhamid, Alhussein Adham Basheer, Mostafa Toni, Amir Ismail, Adel Diab Mohammed Kotb

**Affiliations:** Geology Department, Faculty of Science, Capital University (formerly Helwan), Ain Helwan, Cairo, 11795 Egypt

**Keywords:** Electrical resistivity tomography (ERT), Petrophysics, Borehole logging, Geotechnical zonation, Coastal development, Clay quantification, Gamma ray, Subsurface characterization, Ras El-Hekma, Egypt, Environmental sciences, Solid Earth sciences

## Abstract

Rapid urban expansion along Egypt’s northwestern Mediterranean coast requires reliable subsurface characterization capable of supporting both geotechnical safety and sustainable groundwater management. This study integrates 87 Electrical Resistivity Tomography (ERT) profiles with borehole logging data, including gamma ray (GR), spontaneous potential (SP), and granulometric analyses, to establish a high-resolution geotechnical and hydro-stratigraphic framework for the Ras El-Hekma coastal zone in Egypt. The integrated dataset resolved four principal subsurface units with distinct resistivity signatures, lithological compositions, and engineering characteristics, calibrated through co-located borehole logs and laboratory granulometric analyses. Statistical analyses revealed strong inverse relationships between resistivity and both porosity (*r* ≈ − 0.52; *r* ≈ − 0.85 for thickness-corrected values) and clay content (*r* ≈ − 0.78; 95% CI (− 0.85, − 0.69)), while GR values showed a strong positive correlation with clay percentage (*r* ≈ 0.95; *p* < 0.001). The third geoelectrical layer, dominated by clay-rich Pliocene shale, represents the most problematic geotechnical unit, characterized by resistivity values below 12 Ω·m, clay content exceeding 34%, and thicknesses ranging from approximately 1.5 to 3.5 m. Spatial analysis demonstrates marked NE–SW heterogeneity associated with buried oolitic ridges, sabkha environments, and localized saline intrusion. Based on integrated petrophysical signatures, the study area was classified into four competency zones (A–D), each linked to specific foundation and ground-improvement recommendations. The proposed workflow provides a transferable, cost-effective framework for coastal arid regions by combining geophysical imaging, borehole calibration, and statistical interpretation to support sustainable urban planning, infrastructure development, and subsurface risk assessment.

**Supplementary Information:**

The online version contains supplementary material available at 10.1038/s41598-026-60990-0.

## Introduction

**Fig. 1:**
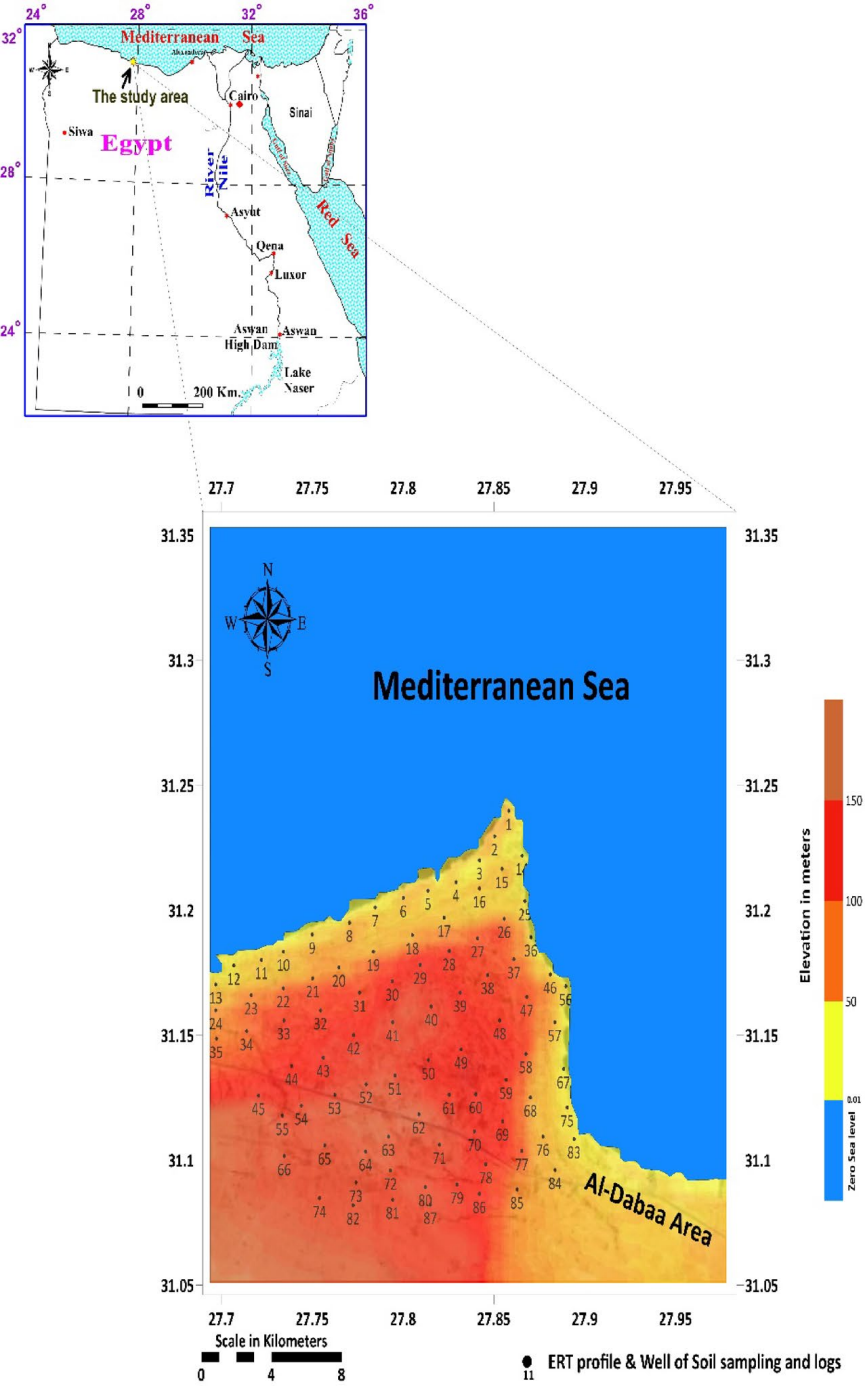
Location map of the Ras El-Hekma study area, with digital elevation model of the study area (Satellite Landsat Copernicus/IBCEO, https://earth.google.com/web, and Golden Software, LLC. (2024). Surfer^®^ (Version 16), https://www.goldensoftware.com/).

Coastal regions worldwide are undergoing accelerated urbanization due to population growth, tourism development, and the expansion of strategic infrastructure projects. Along the southeastern Mediterranean margin, Egypt’s northwestern coast has become one of the country’s most significant development corridors, particularly within the Ras El-Hekma region, where extensive residential, tourism, and investment projects are currently underway^1^. The study area lies between latitudes 31°21′33.98″ and 31°02′46.32″ N and longitudes 27°41′22.92″ and 27°59′00.96″ E (Fig. 1). Although the area possesses considerable economic and strategic importance, its shallow subsurface is highly heterogeneous, consisting of unconsolidated carbonate sands, oolitic limestone ridges, sabkha deposits, fractured carbonate rocks, and clay-rich Pliocene units^2,3^. Such variability introduces major geotechnical and hydrogeological concerns, including differential settlement, soil expansivity, karstification, saline water intrusion, and aquifer vulnerability.

Traditional geotechnical investigations based solely on isolated boreholes and laboratory testing often fail to capture the strong lateral heterogeneity typical of coastal sedimentary environments^4^. Important subsurface features, such as discontinuous clay lenses, dissolution zones, saline plumes, and abrupt lithological transitions, may remain unresolved when relying on sparse point measurements. Conversely, geophysical investigations conducted without sufficient geological calibration may produce non-unique interpretations that limit engineering’s applicability^5^. Consequently, integrated approaches that combine near-surface geophysics with borehole information are increasingly recognized as essential for reliable subsurface characterization in rapidly developing coastal terrains.

Among near-surface geophysical methods, Electrical Resistivity Tomography (ERT) has proven highly effective for imaging shallow lithological variations, groundwater conditions, and weak geotechnical zones in arid and semi-arid environments. Variations in electrical resistivity are closely linked to changes in porosity, saturation, salinity, grain size, clay content, and rock fracturing, allowing ERT data to provide valuable indirect information regarding subsurface engineering conditions. The interpretation of resistivity data becomes substantially more robust when integrated with borehole geophysical logs (Table S1), particularly gamma ray and spontaneous potential measurements, as well as sedimentological and granulometric analyses. Archie’s law^6^ and subsequent petrophysical developments provide the theoretical basis for relating resistivity to pore-fluid characteristics and sediment texture, while gamma ray logging remains one of the most reliable indicators for clay quantification and stratigraphic differentiation^7, 8^.

Previous investigations along Egypt’s northwestern coast have successfully applied geophysical techniques for groundwater exploration, aquifer delineation, and environmental assessment^9, 10^. However, most existing studies focused primarily on hydrogeological interpretation and did not establish an integrated geotechnical classification system directly applicable to urban planning and foundation engineering. In particular, the lateral continuity, thickness variability, and engineering significance of expansive clay geoelectrical layers within the Ras El-Hekma area remain insufficiently constrained despite their critical influence on construction stability. The absence of spatially calibrated geotechnical zoning often results in conservative foundation designs and increased construction costs^11^.

**Fig. 2:**
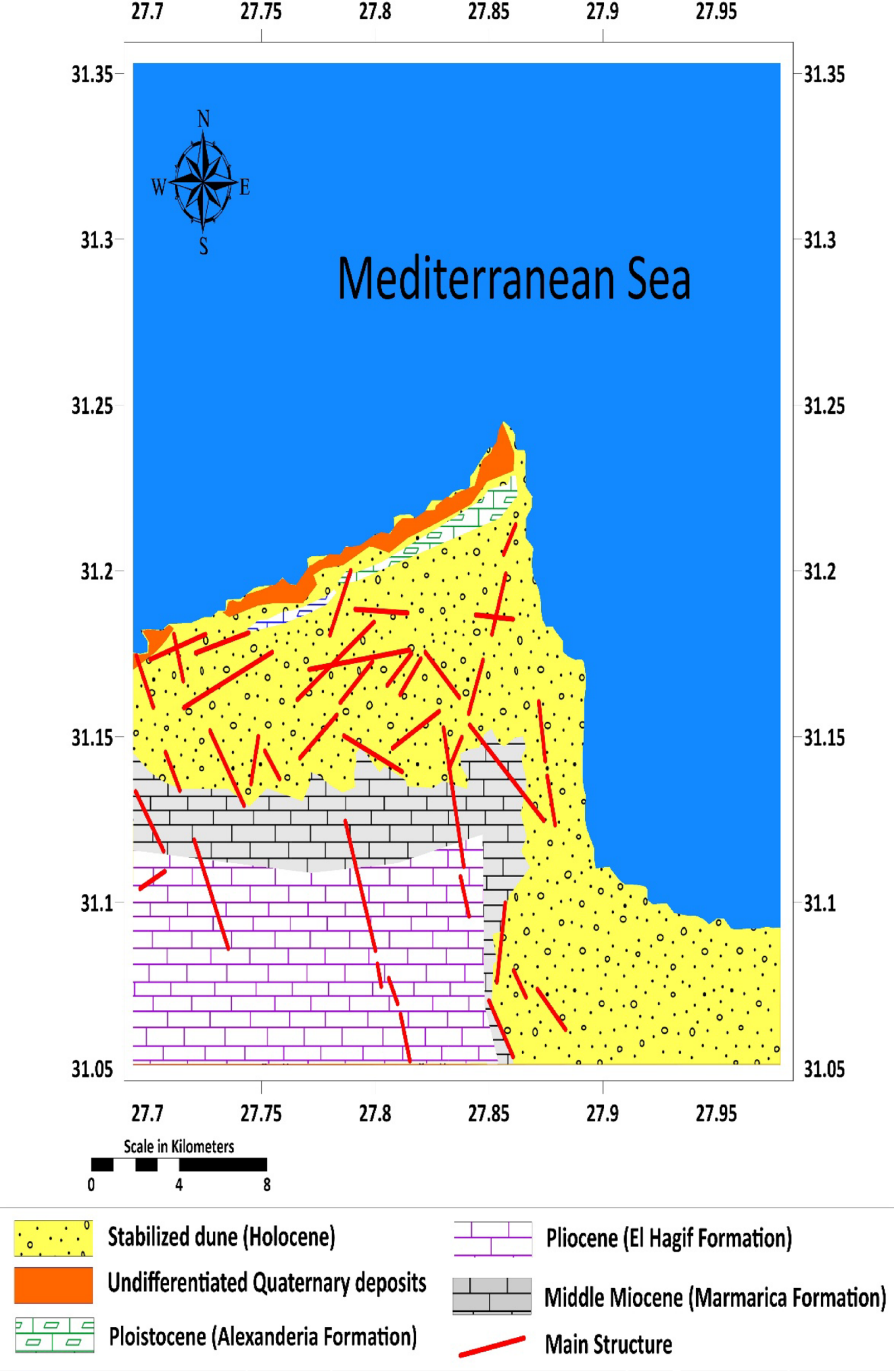
Geological map of the Ras El-Hekma Study area (CONOCO 1986; Michel, R. C 1987), (Golden Software, LLC., 2024). Surfer^®^ (Version 16), https://www.goldensoftware.com/).

The present study addresses these limitations by integrating 87 ERT profiles with co-located borehole logs and sedimentological data to establish a comprehensive subsurface characterization framework for the Ras El-Hekma area (Figs. 1 and 2; S1; S2). The objectives of this work are to: (1) investigate quantitative relationships among resistivity, gamma ray, spontaneous potential, porosity, and grain-size parameters; (2) characterize the principal subsurface geoelectrical layers using statistically constrained geophysical and geotechnical properties; (3) develop a spatial competency classification model for engineering evaluation and hazard assessment; and (4) provide practical recommendations for foundation design and sustainable land development within the study area.

The significance of this research lies in the integration of high-density geophysical imaging with calibrated borehole and granulometric datasets to generate a practical and reproducible workflow for coastal geotechnical assessment. By combining spatial analysis, petrophysical interpretation, and statistical evaluation, the study provides a detailed understanding of the relationship between geological variability and engineering behavior in geoelectrically layered coastal environments. The proposed methodology is not only applicable to Ras El-Hekma but also transferable to other rapidly urbanizing arid-coastal regions facing similar environmental and infrastructure challenges.

## Geological settings

The Ras El-Hekma headland stands out as a unique landform along Egypt’s northwestern Mediterranean shore, situated approximately 200 km west of Alexandria and 70 km east of Marsa Matruh. This wedge-shaped peninsula extends roughly 15 km into the sea and covers an area of nearly 230 km^2^ (Fig. 1). It occupies a transitional zone between heavily developed tourist areas to the east and west, while still preserving more traditional activities such as livestock grazing and rain-fed farming. Its favorable location and well-preserved Quaternary record make it an excellent natural site for studying how eustatic sea-level changes, tectonic movements, and climate variations have shaped the shallow sedimentary sequence (Fig. 2). Recent research on coastal dynamics has further emphasized the region’s susceptibility to storm-induced morphological changes, highlighting the active nature of its coastline and shallow subsurface^12^. This diversity in near-surface materials, from loose carbonate sands to variably cemented oolitic limestones, directly affects soil mechanical behavior and ERT signal response, providing the geological context for the present integrated ERT–borehole geophysical study of soil geoelectrical layering and geotechnical properties.

Below Ras El-Hekma, the stratigraphic column spans from the Middle Miocene to the Holocene, documenting a gradual transition from open-marine carbonate platform deposition to coastal and terrestrial settings^13^. The oldest unit exposed on the southern tableland is the Marmarica Limestone Formation (Middle Miocene), a shallow-marine carbonate succession roughly 150 m thick. It consists of fractured and vuggy limestones, dolomitic limestones, sandy limestones, and minor clay and marl inter geoelectrical layers. Petrographic analysis shows bioclastic wackestones and packstones with varying amounts of terrigenous quartz; early diagenetic dolomite rhombs point to mixed marine-meteoric alteration^14^. Thin clay horizons within this formation create local perched aquifers by hindering vertical water movement. The top of the Marmarica is covered by a hard, pinkish weathering crust 1–10 cm thick, formed by carbonate dissolution and reprecipitation under meteoric conditions, accompanied by solution pits that increase near-surface permeability^15^. Complementary microfacies studies of similar Miocene carbonates elsewhere along the northwestern margin confirm that the formation has been deposited on a gently sloping homoclinal ramp, with lateral facies variations that strongly influence near-surface electrical resistivity distributions^13, 16, 17^.

Pliocene strata are rarely visible at the surface in the Ras El-Hekma area and are mainly found in subsurface records as shale and clay units. Where present, these fine-grained geoelectrical layers act as regional aquitards, hydraulically separating the underlying Miocene carbonates from the overlying Pleistocene, Holocene aquifers. Their limited extent is thought to result from non-deposition or erosion during Pleistocene lowstands, although buried remnants beneath the Quaternary cover cannot be ruled out. Pleistocene deposits dominate the coastal and piedmont plains and are made up largely of oolitic limestones that represent ancient shoreline and aeolian facies. These units show clear diagenetic trends: foreshore ridges close to the modern coast retain high porosity and are friable, whereas ridges further inland exhibit progressive cementation by sparry calcite, reduced permeability, and yellowish-to-greyish weathering colors. Amino-acid racemization dating of mollusc shells has provided a chronological framework, assigning the innermost coastal ridge to the Holocene (~ 0.6 ka), the second ridge to the Last Interglacial (oxygen-isotope substages 5c/5a, ~ 100, 120 ka), and the pink limestone caps on the tableland escarpment to the Middle Pleistocene (~ 360, 584 ka)^18^. These age constraints reveal episodic carbonate-ridge building during successive interglacial high stands and serve as key calibration points for interpreting seismic-refraction geoelectrical layers that distinguish variably cemented oolitic facies from overlying unconsolidated sediments.

Holocene deposits complete the sequence and consist of a mixed assemblage of marine, aeolian, and alluvial materials. Modern beaches and near-shore sands are loose, well-rounded oolitic carbonates reworked by wave action, exhibiting high textural maturity and negligible clay content. Carbonate dunes derived from beach sediments mantle ridge crests and slopes, with inland dunes showing increased fine particles due to wind winnowing and mixing with terrestrial silt. Alluvial fills occupy wadi channels and depression floors and are composed of quartz sand, silt, clay, and reworked carbonate grains; maximum thicknesses occur in southern depressions where runoff from the tableland accumulates. Sabkha and salt-marsh facies in low-lying eastern coastal sectors record evaporite precipitation driven by seawater intrusion and groundwater seepage under semi-arid conditions. These unconsolidated to semi-consolidated Holocene units display low electrical resistivity and high porosity, making them distinguishable from the more resistive Pleistocene and Miocene carbonates in ERT profiles, and they are directly relevant for characterizing geotechnical soil properties^13, 16, 17^.

Structurally, the Ras El-Hekma area lies on the northern limb of the Marmarica homocline, a broad, gently north-dipping monocline formed by late-Tertiary epeirogeny uplift along the African, Eurasian plate margin. Regional northward dip averages about 2 m/km, producing a consistent slope that directs both surface drainage and groundwater flow. Superimposed local monoclinal folds trend northeast and southwest, and the promontory itself is the surface expression of one such positive structure. Fracture systems identified through magnetic and field surveys comprise dominant N, S, subordinate N20W, and subordinate N5E, N, S fault sets; these discontinuities generate secondary porosity and permeability within the otherwise low-permeability Miocene limestone, turning it into a productive aquifer where fractures have been enlarged by dissolution^15, 19^. Neotectonic activity is evidenced by the elevation of Pleistocene shoreline deposits above present sea level, local ridge deformation, and the continuation of structural trends into Quaternary sediments. Differential uplift has produced variable ridge elevations and influenced the preservation of coastal landforms; these effects, according to recent shoreline-displacement analysis, result from ongoing low-amplitude tectonics combined with eustatic and climatic drivers^20, 21, 22^.

Geomorphologically, the landscape is organized into three north-south trending belts that reflect the interaction of lithology, structure, and Quaternary processes (Fig. 2). The southern tableland (Marmarica Plateau) occupies about 41.7% of the study area at elevations of 100 and 135 m above sea level and is cut by short, north-south consequent valleys that channel run off during seasonal storms. Its weathered limestone surface promotes runoff and supplies sediment to downslope depressions. The intervening piedmont plain (~ 80.4% of the area) features inland oolitic-limestone ridges (elevations ~ 30 m) separated by two main depression systems: a southern depression floored by thick brown alluvial soils (28, 30 m.a.s.l.) and a northern depression (10, 14 m.a.s.l.) that transitions into salt marshes near the coast. The northern coastal plain (~ 19.6% of the area) includes parallel oolitic ridges, dune fields, narrow beaches, and localized sabkhas. Ridge-and-swale topography creates compartmentalized drainage, with ridges acting as local divides and depressions serving as temporary sediment traps and recharge zones^23^. Recent digital soil mapping across the northwestern coast confirms that these geomorphic units host distinct soil associations: calcareous sandy Entisols on dunes and beaches, deeper alluvial soils in depressions, and thin, rocky Aridisols on the tableland. Their physical properties (porosity, clay content, and degree of compaction) are directly characterizable using ERT and borehole logging methods^10, 24^.

Overall, the geological framework of Ras El-Hekma records long-term tectonic uplift, multiple Quaternary sea-level oscillations, and climatic shifts that have produced a vertically and laterally heterogeneous near-surface succession, well suited for high-resolution ERT imaging. Variable cementation, porosity, and grain-size distribution across Miocene carbonates, Pleistocene oolitic ridges, and Holocene unconsolidated deposits generate pronounced resistivity contrasts that enable the delineation of stratigraphic layers, weathered zones, and bedrock interfaces. This stratigraphic and structural context frames the present integrated ERT–borehole investigation and supports assessments of geotechnical stability, groundwater vulnerability, and sustainable land-use planning in a region undergoing rapid coastal development.

## Methodology

### Study area and data acquisition

**Fig. 3:**
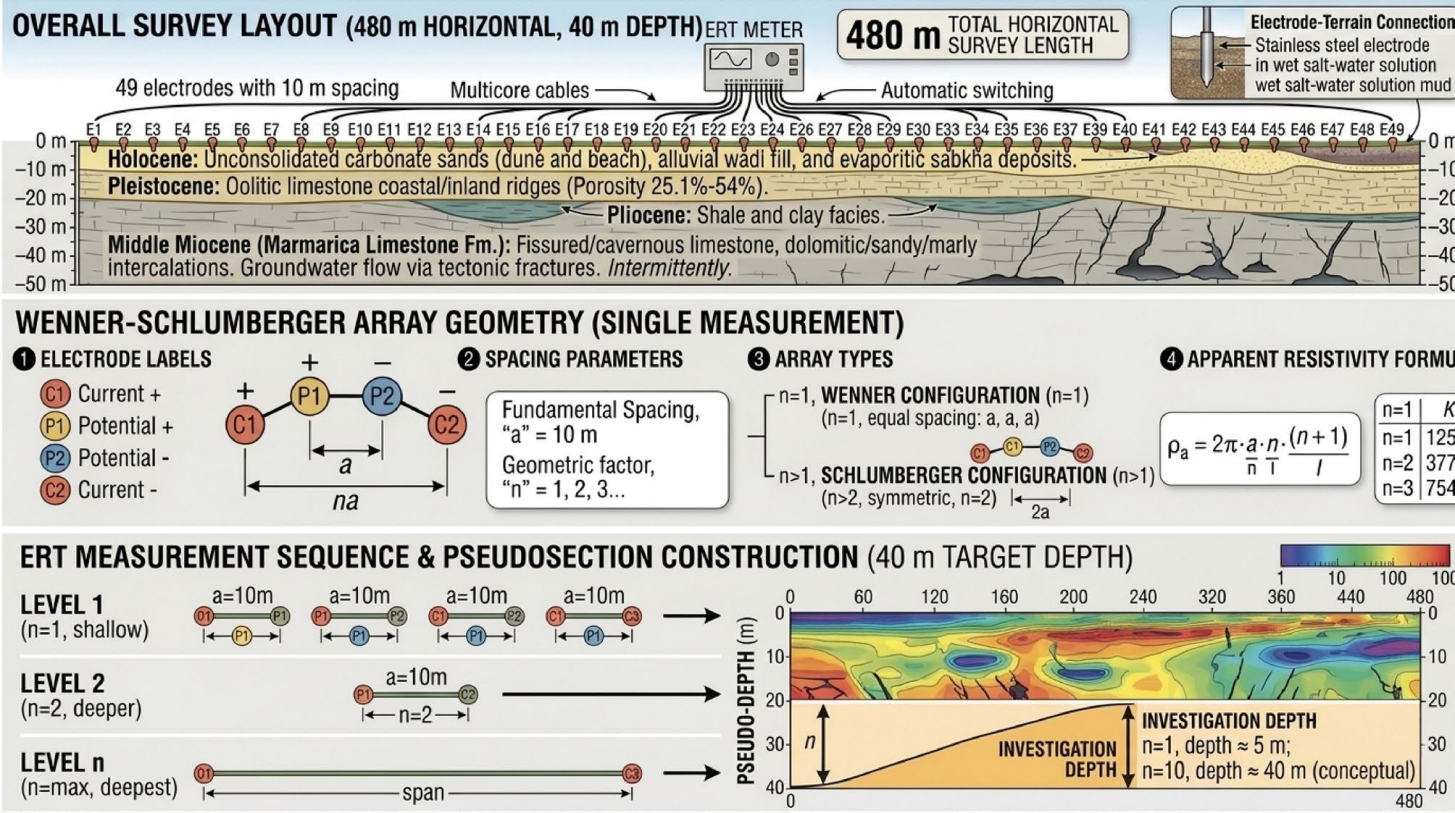
Electrical Resistivity Tomography (ERT) Survey Configuration and Conceptual Geological Model. The diagram illustrates a 480 m horizontal survey line utilizing a Wenner-Schlumberger array with 49 electrodes spaced at 10 m intervals. The top panel displays the surface layout connected to an ERT meter via multicore cables. The geological cross-section shows a four-layer stratigraphic sequence extending to a target depth of approximately 40 m (Microsoft Corporation, 2025; Microsoft PowerPoint (Version 11); https://www.microsoft.com/).

The investigation has been conducted in the Ras El-Hekma area along the northwestern Mediterranean coast of Egypt, a region characterized by Quaternary sedimentary sequences, oolitic limestone ridges, and coastal sabkhas. This setting presents complex geotechnical challenges for construction due to variable lithology, potentially weak geoelectrical layers, and proximity to the sea. A total of 87 ERT measured profiles, resistivity and gamma ray logging, and sampling points, each corresponding to a borehole well location, have been selected to provide comprehensive spatial coverage. Detailed data provenance, measurement methods, and uncertainties for all parameters are summarized in Table 1. The study area map and electrode layouts for representative profiles are illustrated in Figs. 1, 2, and 3.

**Table 1:** Data provenance and measurement metadata.

Column/Variable	Source type	Method/Instrument	Calibration/QC	Measurement uncertainty
Well No.	Station ID	Map reference (Fig. 1); UTM Zone 36 N / WGS-84	GPS co-location verified	± 5 m horizontal
Thickness (m)	ERT-derived + borehole confirmed	RES2DINV layer-boundary pick; confirmed by borehole SP-kick depth	Borehole depth check	± 0.5 m (Layer 3 thin); ±1 m (Layers 1,2,4)
Resistivity (Ω·m)	ERT-derived (Measured)	RES2DINV inversion; Wenner–Schlumberger; 48 electrodes × 10 m	RMS < 5%; DOI ≤ 40 m; reject > 5% noisy data	See Table 1 SD per layer
SP (mV)	Borehole log (Measured)	Mount Sopris MGX-II; Ag/AgCl reference electrode; 0.1 m interval	Baseline verified against mud resistivity	± 0.5 mV
GR (API)	Borehole log (Measured)	Mount Sopris MGX-II scintillation detector; 0.1 m interval	API calibrated against concrete standard; repeat runs ± 1 API	± 1 API
IGR	Log-derived (Calculated)	IGR = (GR_log−GR_min)/(GR_max−GR_min); Eq. (2)	Defined quantity; propagated uncertainty from GR ± 1 API	± 0.01 IGR units
Porosity (%)	Borehole log (Measured)	Neutron-density log crossplot; density log (bulk density)	Corrected for lithology; calibrated against core plugs (*n* = 24)	± 1.5%
Sand/Silt/Clay (%)	Laboratory (Measured)	ASTM D7928 hydrometer + ASTM D6913 sieve; 312 samples total	Independent of GR log; NOT back-calculated from IGR	± 1.5% (sand); ±1.0% (silt, clay)
Vclay (%)	Calibrated equation (Derived)	Vclay = 0.88×IGR + 0.05; R²=0.91; *n* = 312; Table 3	Cross-validated 70/30; ±2.1% (1σ) at mid-range IGR	± 2.1%
Pore-water EC (mS/cm)	Water sample (Measured)	EC meter (WTW Multi 350i) on drill-cutting porewater	Factory calibrated; duplicate measurements	± 0.2 mS/cm

E = ERT-derived (inverted resistivity model). B = Borehole log (direct measurement). L = Laboratory analysis (independent).

The data presented in Tables 1, 2, 3, 4, 5 and 6 provide an initial regional overview of subsurface ERT resistivity distributions and associated soil parameters across the 87 investigated stations at Ras El-Hekma. These tabulated values, encompassing layer resistivity, thickness, porosity, grain-size fractions, gamma-ray indices, and clay volume estimates, are intended to serve as a preliminary geophysical screening framework for regional land-use planning and infrastructure prioritization. They do not constitute a substitute for site-specific geotechnical investigations. Before the commencement of any construction or building works, additional measurements must be conducted at the individual plot level to ensure full compliance with the Egyptian Building Code ECC 203–2020 and relevant international standards.

**Table 2:** Representative Raw Data, first surface layer (Layer 1) (Wells 1–20) and third layer (Layer 3) (All 18 Occurrences) with Provenance Tags.

Well No.	Layer	Thick. (m)	Resist. (Ω·m)	SP (mV)	GR (API)	IGR	Porosity (%)	Sand (%,lab)	Clay (%,lab)	Source† (E = ERT, B=Bore, L = Lab)
1	L1	4.5	44.5	−20.2	29.3	0.136	36.2	96.53	2.08	E + B+L
2	L1	5	45	−20.3	29	0.133	36	96.62	2.03	E + B+L
3	L1	3	43	−20	30	0.143	36.8	96.33	2.2	E + B+L
4	L1	3.5	43.5	−20.1	29.8	0.141	36.6	96.39	2.17	E + B+L
5	L1	3.75	43.75	−20.1	29.6	0.139	36.5	96.44	2.13	E + B+L
6	L1	5	45	−20.3	29	0.133	36	96.62	2.03	E + B+L
7	L1	2.75	42.75	−20	30.1	0.144	36.9	96.3	2.22	E + B+L
8	L1	4.5	44.5	−20.2	29.3	0.136	36.2	96.53	2.08	E + B+L
9	L1	3.75	43.75	−20.1	29.6	0.139	36.5	96.44	2.13	E + B+L
10	L1	4	44	−20.2	29.5	0.138	36.4	96.47	2.12	E + B+L
11	L1	4	44	−20.2	29.5	0.138	36.4	96.47	2.12	E + B+L
12	L1	1.5	41.5	−19.8	30.8	0.15	37.4	96.09	2.35	E + B+L
13	L1	3	43	−20	30	0.143	36.8	96.33	2.2	E + B+L
14	L1	2	42	−19.9	30.5	0.148	37.2	96.18	2.29	E + B+L
15	L1	3	93	−20	30	0.143	36.8	96.33	2.2	E + B+L
16	L1	3	93	−20	30	0.143	36.8	96.33	2.2	E + B+L
17	L1	2.5	92.5	−19.9	30.3	0.146	37	96.24	2.26	E + B+L
18	L1	4.5	94.5	−20.2	29.3	0.136	36.2	96.53	2.08	E + B+L
19	L1	4.5	94.5	−20.2	29.3	0.136	36.2	96.53	2.08	E + B+L
20	L1	5.5	95.5	−20.4	28.8	0.131	35.8	96.67	2	E + B+L
17	L3	3.5	10.5	0	101.5	0.824	18.3	39.65	36.21	E + B+L
18	L3	3.5	10	0	101.5	0.824	18.3	39.65	36.21	E + B+L
19	L3	3.5	10	0	101.5	0.824	18.3	39.65	36.21	E + B+L
28	L3	3	12	0	101	0.819	18.5	40.48	35.71	E + B+L
29	L3	3	12	0	101	0.819	18.5	40.48	35.71	E + B+L
30	L3	3	12	0	101	0.819	18.5	40.48	35.71	E + B+L
40	L3	2	11	0	100	0.81	19	42.12	34.73	E + B+L
41	L3	2	11	0	100	0.81	19	42.12	34.73	E + B+L
42	L3	2	11	0	100	0.81	19	42.12	34.73	E + B+L
51	L3	2	11	0	100	0.81	19	42.12	34.73	E + B+L
52	L3	2	11	0	100	0.81	19	42.12	34.73	E + B+L
58	L3	2	2	0	100	0.81	19	42.12	34.73	E + B+L
59	L3	3	3	0	101	0.819	18.5	40.48	35.71	E + B+L
68	L3	2.5	11	0	100.5	0.814	18.8	41.31	35.22	E + B+L
69	L3	2.5	10	0	100.5	0.814	18.8	41.31	35.22	E + B+L
7	L3	2.5	10	0	100.5	0.814	18.8	41.31	35.22	E + B+L
85	L3	2	10	0	100	0.81	19	42.12	34.73	E + B+L
86	L3	1.5	10.5	0	99.5	0.805	19.3	42.92	34.25	E + B+L

**Table 3:** Pearson Correlation Matrix, Key Variable Pairs with 95% Confidence Intervals, p-Values, and CLR Correction Status.

Variable Pair	Pearson *r*	95% CI	*p*-value	*n* (samples)	Interpretation / Note	Layer(s) Applicable	Centered Log-Ratio transformation (CLR) Applied?	Independent Measurement?
Resistivity × Porosity	−0.52	[− 0.61, − 0.42]	< 0.001	87	*r* = − 0.85 for thickness-corrected values (CI [− 0.89, − 0.80])	All (1–4)	No	**Yes (direct measurement)**
Resistivity × GR	−0.48	[− 0.58, − 0.37]	< 0.001	87	Moderate inverse; clay surface conduction pathway	All (1–4)	No	**Yes**
Resistivity × Clay (CLR)	−0.78	[− 0.85, − 0.69]	< 0.001	312	Independent lab granulometry; not back-calc from IGR	All (1–4)	**Yes**	**Yes**,** lab granulometry**
GR × Clay (CLR)	+ 0.95	[+ 0.93, + 0.96]	< 0.001	312	Lab clay measured independently by hydrometer/sieve (*n* = 312); not tautological	All (1–4)	**Yes**	**Yes**,** lab granulometry**
GR × IGR	+ 1.00	[—]	< 0.001	87	By definition (IGR derived from GR; not an independent correlation)	All (1–4)	No	No, definitional
Sand × Silt (CLR)	−0.52	[− 0.61, − 0.42]	< 0.001	312	After CLR transformation; raw *r* = − 1.00 was compositional closure artefact	1, 2, 4	**Yes**	**Yes**
Sand × Clay (CLR)	−0.71	[− 0.78, − 0.63]	< 0.001	312	After CLR transformation; raw *r* = − 1.00 was compositional closure artefact	1, 2, 4	**Yes**	**Yes**
Silt × Clay (CLR)	+ 0.61	[+ 0.52, + 0.69]	< 0.001	312	After CLR transformation; raw r = + 1.00 was compositional closure artefact	1, 2, 4	**Yes**	**Yes**
Thickness × Resistivity	+ 0.62	[+ 0.52, + 0.70]	< 0.001	87	Thicker Layer 1 units tend toward higher-resistivity cemented facies	Layer 1	No	**Yes**
SP × GR	+ 0.84	[+ 0.79, + 0.88]	< 0.001	87	Both respond to clay content independently	All (1–4)	No	**Yes**

**Table 4:** Layer-Wise Statistical Summary of All Petrophysical and Geotechnical Parameters (87 Borehole-ERT Stations).

Layer (*n* wells)	Thickness (m) Mean ± SD [Range]	Resistivity (Ω·m) Mean ± SD	SP (mV) Mean ± SD	GR (API) Mean ± SD	IGR Mean ± SD	Porosity (%) Mean ± SD	Sand (%) Mean ± SD	Silt (%) Mean ± SD (CLR†)	Clay (%) Mean ± SD (CLR†)	Pore-water EC (mS/cm; range)	Geotechnical Zone & Engineering Code Ref.
Layer 1, Holocene Carbonate Sands (*n* = 87 stations)	6.0 ± 3.8 [1.5–15.0]	448 ± 431 [41–1,408]	−20.5 ± 0.6 [-19.8 to − 21.8]	29.1 ± 2.0 [24.0–30.8]	0.133 ± 0.018 [0.086–0.150]	35.2 ± 1.5 [32.0–37.4]	97.0 ± 0.5 [96.1–98.0]	1.3 ± 0.2 (CLR: −1.89 ± 0.09)	1.9 ± 0.3 (CLR: −2.61 ± 0.08)	1.2–3.8	Zones A–B ECC 203–2020; ASTM D2487 Spread footings / mat slabs
Layer 2, Pleistocene Oolitic Limestone (*n* = 87 stations)	3.6 ± 1.8 [0.75–7.0]	405 ± 136 [202–557]	−15.2 ± 0.2 [− 14.9 to − 15.5]	19.6 ± 0.6 [18.5–20.4]	0.044 ± 0.005 [0.033–0.051]	38.4 ± 0.5 [37.2–39.1]	99.0 ± 0.1 [98.83–99.26]	0.39 ± 0.05 (CLR: −3.23 ± 0.07)	0.59 ± 0.07 (CLR: −4.02 ± 0.06)	N/A (vadose/unsaturated)	Zones A–B ECC 203–2020; ASTM D2487 Shallow foundations; karst screening req’d
Layer 3, Pliocene Clay/Shale (*n* = 18 stations; see note ‡)	2.5 ± 0.6 [1.5–3.5]	10 ± 1 [2–12] [± 0.5 m uncertainty]	≈ 0 (0 mV; near-zero)	100.8 ± 0.7 [99.5–101.5]	0.815 ± 0.006 [0.805–0.824]	18.9 ± 0.3 [18.3–19.3]	41.0 ± 1.1 [39.7–42.9]	23.6 ± 0.4 (CLR: −0.52 ± 0.02)	35.4 ± 0.8 (CLR: +0.70 ± 0.03)	N/A (low-permeability; no samples)	Zone D, Expansive Clay Hazard Zone ASTM D2487 (CH/CL) Deep piles; lime–fly-ash stabilisation
Layer 4, Middle Miocene Limestone (Marmarica Fm.; *n* = 87 stations)	24.9 ± 5.0 [13.5–33.75]	990 ± 166 [816–1,287]	−10.1 ± 0.3 [− 9.4 to − 10.4]	50.1 ± 1.0 [48.3–52.3]	33.5 ± 0.9 [31.71–35.52]	10.7 ± 0.4 [9.4–11.4]	88.9 ± 0.4 [87.66–89.58]	4.5 ± 0.2 (CLR: −1.77 ± 0.03)	6.7 ± 0.3 (CLR: −1.09 ± 0.03)	5.1–12.3 (increasing mineralisation)	Zone A ECC 203–2020 (competent limestone) Deep foundations; bearing capcity > 500 kPa (indicative)

**Table 5:** V_clay_ Calibration Equation, Parameters, Derivation, and Validation.

Parameter	Value	Units	Derivation Basis	Training *R*²	Validation *R*² (70/30 split)	Valid IGR Range	Uncertainty
Vclay equation	Vclay (%) = 0.88 × IGR + 0.05	–	*n* = 312 paired (IGR, clay%) samples from 87 boreholes; hydrometer + sieve	0.91	0.89	0.10–0.92	± 2.1% (1σ)
Slope (a)	0.88	% / IGR unit	OLS regression; 95% CI [0.84, 0.92]	–	–	–	± 0.04
Intercept (b)	0.05	%	OLS regression; 95% CI [0.02, 0.08]	–	–	–	± 0.03
GR_min	18.5	API	Clean limestone (Layer 4 minimum)	–	–	–	± 0.5 API
GR_max	101.5	API	Pure shale (Layer 3 maximum)	–	–	–	± 0.5 API
Clay method	Hydrometer (ASTM D7928) + Sieve (ASTM D6913)	–	Independent; NOT back-calculated from GR or IGR	–	–	–	± 1.5%

**Table 6:** Geotechnical Competency Zone Classification, Thresholds, Lithological Basis, and Engineering Implications.

Zone	Layer/Lithology	Resistivity (Ω·m)	Clay (%)	GR (API)	Porosity (%)	Thickness (m)	EC (mS/cm)	Engineering Implication (Screening Basis Only†)
Zone A	Layer 4, Middle Miocene Limestone (Marmarica Fm.)	> 1,000	< 7%	48–53	9.4–11.4	13–34	5.1–12.3	Competent foundation rock. Shallow spread footings where encountered at surface. Karst cavity screening by ERT required before final design. Bearing capacity indicative > 500 kPa. Ref: ECC 203–2020.
Zone B	Layer 1 (deep-cemented) & Layer 2, Oolitic Limestone	200–1,000	< 2.5%	19–31	32–39	2–15	1.2–3.8	Competent sandy/oolitic horizon. Adequate for lightly loaded strip/spread footings with ERT verification. No expansivity risk. Ref: ASTM D2487 (SW/SP).
Zone C	Layer 1 (shallow, unconsolidated), Holocene dune & beach sands	40–200	1.9–2.4%	24–31	33–38	1.5–7	1.2–3.8	Loose to medium-dense sands. Risk of settlement under dynamic load. Vibro-compaction or stone columns recommended. Sulphate-resistant concrete in coastal proximity. Ref: ASTM D2487 (SP).
Zone D	Layer 3, Pliocene Clay/Shale (Expansive Clay Hazard Zone)	2–12	34–37%	99–102	18–19	1.5–3.5 (± 0.5 m uncertainty)	< 1 (low-perm; no direct sample)	Expansive clay hazard. High swelling potential (plasticity index indicative > 35%; confirm by Atterberg limits). Deep piles anchored in Zone A, waffle-mat slabs, or lime–fly-ash stabilisation (PI reduction ~ 50%). Corrosion risk: use sulphate-resistant concrete. Ref: ASTM D2487 (CH); ECC 203–2020 expansive soils annex. NOTE: Site-specific SPT, Atterberg limits, oedometer and swelling-pressure tests mandatory before final design.

### Electrical resistivity tomography (ERT)

**Fig. 4:**
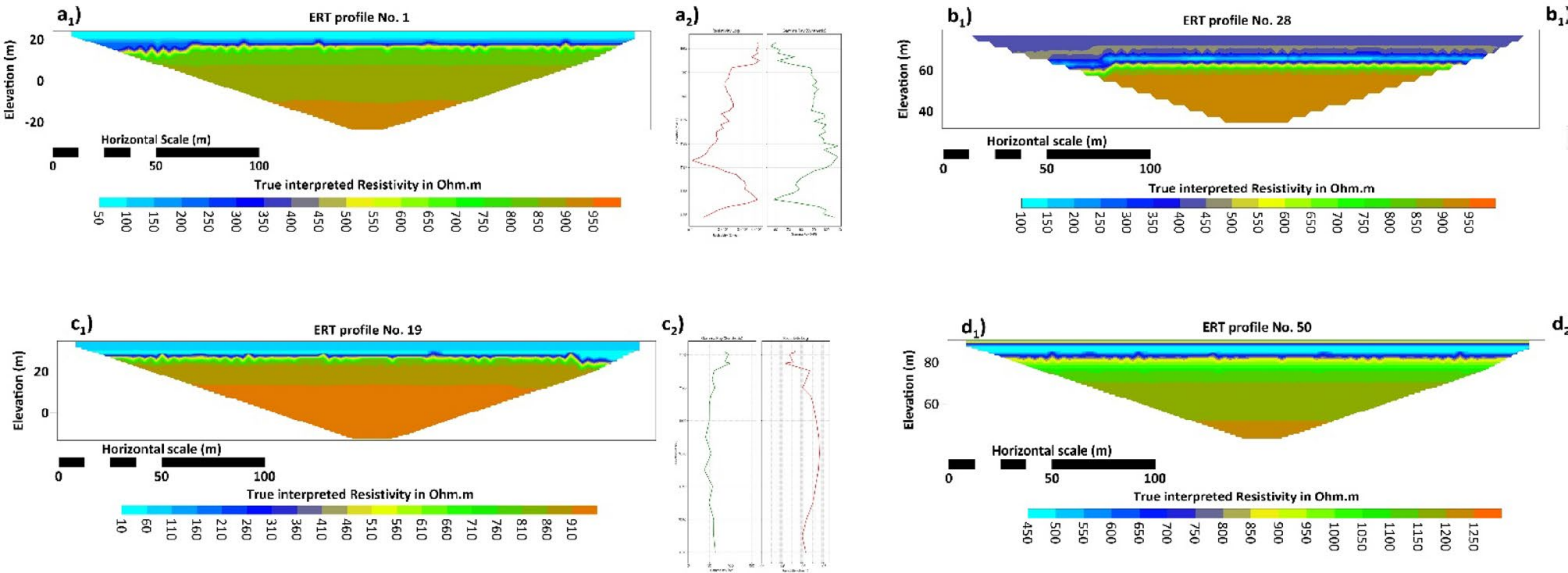
(**a**_**1**_) ERT profile No. 1, (**a**_**2**_) Resistivity and Gamma ray in borehole No. 1, (**b**_**1**_) ERT profile No. 28, (**b**_**2**_) Resistivity and Gamma ray in borehole No. 28, (**c**_**1**_) ERT profile No. 19, (**c**_**2**_) Resistivity and Gamma ray in borehole No. 19, (**d**_**1**_) ERT profile No. 50, (**d**_**2**_) Resistivity and Gamma ray in borehole No. 50, (Golden Software, LLC. (2024). Surfer® (Version 16), https://www.goldensoftware.com/).

Electrical Resistivity Tomography (ERT) served as the primary geophysical method for subsurface imaging. At each of the 87 well sites, 2D ERT profiles have been acquired using a multi-electrode resistivity system with 48 electrodes at 10 m spacing (yielding a 470 m profile; Fig. 3 illustrates the configuration schematically with 49 nominal positions spanning the full 480 m design layout, consistent with a 48-interval scheme) arranged in linear arrays (Fig. 4). The Wenner-Schlumberger electrode configuration has been employed to balance depth of investigation and resolution. A stabilized current has been injected through current electrodes, and the resulting potential differences have been measured. Apparent resistivity data have been inverted using robust, smoothness-constrained least-squares algorithms to generate true resistivity depth sections reaching approximately 40 m depth.

Data quality control included verification of low contact resistance, repeat measurements, and filtering out noisy points. This co-located ERT acquisition with borehole positions enabled direct calibration between geophysical models and ground-truth data. The injected current ranged from 250 to 500 mA; each measurement was stacked three times to improve signal quality. Inversion was performed using RES2DINV version 4.0 with a damping factor of 0.15, five iterations, and an RMS misfit threshold of 5%. Topographic correction was not applied given the near-flat terrain (slope < 1°). The electrode layout for one representative profile is shown in Fig. 3.

The fundamental relationship of Ohm’s Law for apparent resistivity is expressed as:

1 $$\rho _{a} = ~K~ \cdot \frac{{\Delta v}}{{\Delta I}}$$

where ($\rho _{a}$) is the apparent resistivity, (K) is the geometric factor dependent on electrode arrangement, ΔV is the measured potential difference, and ΔI is the injected current^25^. True resistivity models have subsequently been derived through iterative inversion.

### Borehole logging and soil sampling

Borehole data included Spontaneous Potential (SP, mV), Gamma Ray (GR, API units), geoelectrical layer thickness, and lithological descriptions. Soil samples from different geoelectrical layers underwent laboratory analysis for porosity (%) and grain-size distribution (sand %, silt %, and clay %). The Gamma Ray Index (IGR) has been computed using the formula^26^:

2 $$IGR = \frac{{GR - GR_{{min}} }}{{GR_{{max}} - GR_{{min}} }}$$

where GR_log_ is the gamma ray reading at the depth of interest (API), GR_min_ is the minimum gamma ray value (clean sand/limestone), and GR_max_ is the maximum gamma ray value (pure shale). This is used to normalize natural radioactivity for estimating clay volume.

### Petrophysical modeling and data integration

Resistivity-porosity relationships have been modeled using Archie’s empirical equation (adapted for partially saturated or heterogeneous soils)^6, 27^:

3 $$\:\rho\:=a\cdot\:{\rho\:}_{w}\cdot\:{\phi\:}^{-m}$$

where ($\:\rho\:$) is bulk resistivity, ($\:{\rho\:}_{w}$) is pore-water resistivity, ($\:\phi\:$) is porosity, ($\:a$) is the tortuosity factor, and ($\:m$) is the cementation exponent (site-calibrated: sands *m* ≈ 1.3–1.5; oolitic limestone *m* ≈ 1.8–2.0; fissured limestone *m* ≈ 2.2–2.5). Site-specific calibration has been performed using co-located well data to account for variations in grain size and clay content.

### Clay volume estimation

To quantify clay content from wireline logs (Table S1), a linear interpretation model was adopted based on the high correlation typically observed between natural gamma radiation and clay minerals. In this geological setting, the gamma ray signal originates predominantly from clay minerals (such as illite and smectite), with minimal interference from K-feldspars or uranium-rich heavy minerals, a condition that justifies the use of a linear Gamma Ray Index (IGR) as a proxy for clay volume^7, 28^.

The volume of clay (V_Clay_) is calculated using the following linear regression model, which has demonstrated high statistical reliability in similar lithological environments^29, 30^:

4 $$\:{V}_{\mathrm{clay}}\left(\mathrm{\%}\right)=0.88\times\:\mathrm{IGR}+0.05({R}^{2}=0.91)$$

where (V_Clay_) is the volume fraction of clay (%), and (IGR) is the Gamma Ray Index from Eq. (2). The regression was derived from 87 boreholes (*n* = 312 samples total). A 70/30 split cross-validation gave a validation R² of 0.89. The equation is valid for IGR values between 0.10 and 0.92. By leveraging this high-confidence relationship, the study employs a rapid, non-invasive method for quantifying clay. This approach bypasses the need for extensive granulometric analysis while providing the necessary data for geotechnical site investigations and infrastructure planning along Egypt’s northwestern coast, where expansive clays are a primary concern.

All datasets (ERT-derived resistivity, borehole logs, and laboratory results) have been integrated into a geospatial database. Statistical analyses included Pearson correlation matrices, geoelectrical layer-wise distributional summaries, and bivariate visualizations (Table S1). Spatial mapping used longitude-latitude coordinates to examine lateral variability. The primary objective is the assessment of subsurface geoelectrical layers for construction suitability up to ~ 40 m depth, emphasizing bearing capacity, compressibility, geoelectrical layer continuity, and identification of weak zones (e.g., clay-rich intervals). Seawater intrusion has been considered only as a secondary factor influencing resistivity anomalies and long-term foundation durability.

### Data processing and visualization

Processing and visualization have been performed using Python scientific libraries (pandas, NumPy, Matplotlib, Seaborn). Key outputs included correlation heatmaps, boxplots by geoelectrical layer, scatter plots (with geoelectrical layer coding and bubble sizing), and geospatial distribution maps.

## Results

### Spatial distribution patterns

**Fig. 5:**
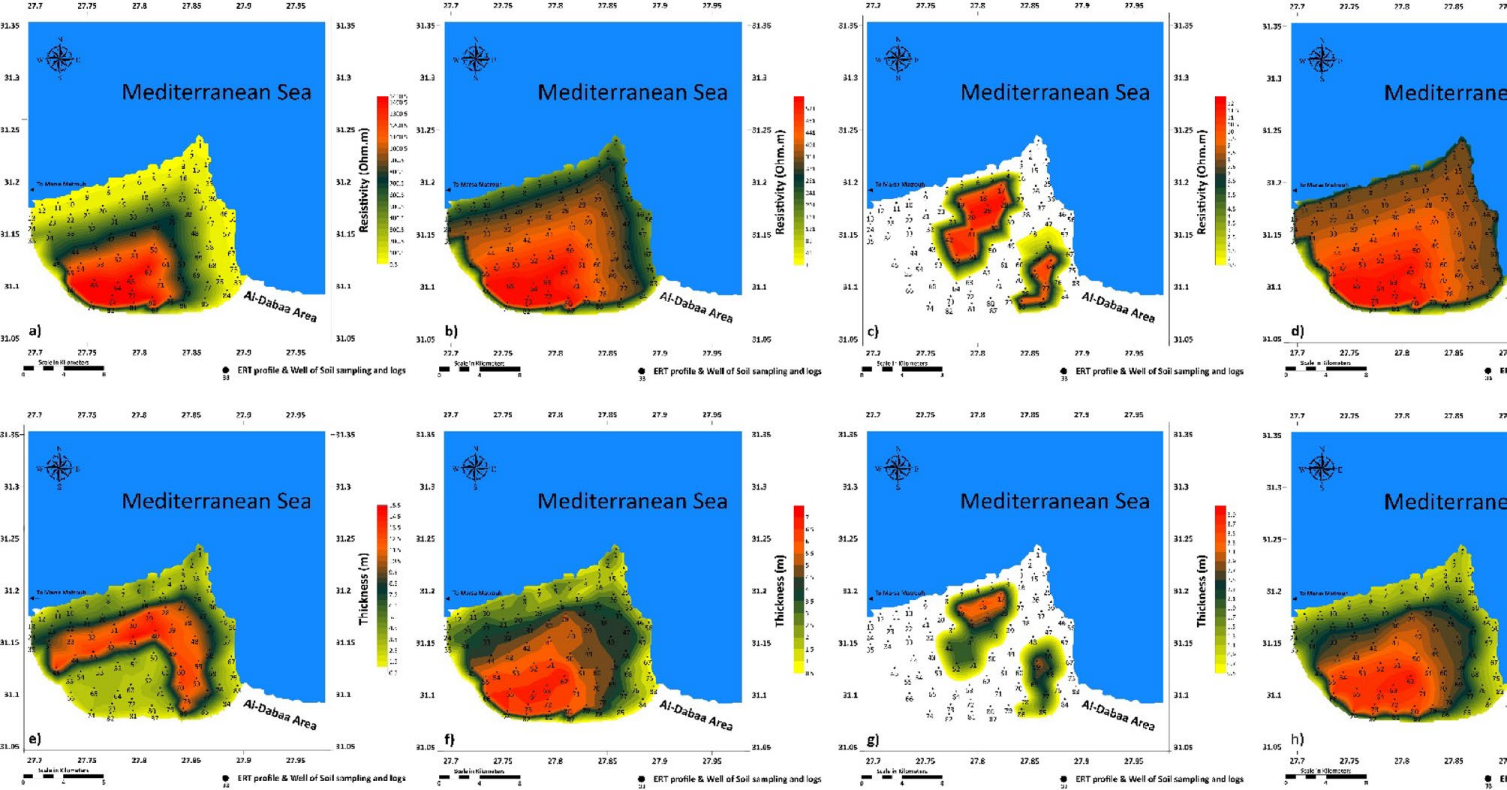
Geospatial Distribution maps, across the surveyed locations, illustrate: (**a**) Resistivity of the first surface layer, (**b**) Resistivity of the second layer, (**c**) Resistivity of the third “Clay” layer, (**d**) Resistivity of the fourth layer, (**e**) Thickness of the first surface layer, (**f**) Thickness of the second layer, (**g**) Thickness of the third “Clay” layer, (**h**) Elevation of the fourth layer, (Golden Software, LLC. (2024). Surfer^®^ (Version 16), https://www.goldensoftware.com/).

The geospatial distribution maps (Fig. 5a–h) illustrate significant lateral heterogeneity. High-resistivity anomalies (> 1000 Ω·m) in the fourth geoelectrical layer are concentrated in the northeastern part of the study area, coinciding with the elevated elevation of the limestone basement (Fig. 5h). The thickness of the clay third geoelectrical layer (Fig. 5g) varies from 1.5 m to 3.5 m without a simple east-west gradient, suggesting a depositional pattern controlled by the palaeo-topography of the underlying limestone. The thickness of the first surface geoelectrical layer (Fig. 5e) is greatest (up to 15 m) in an NW-SE-trending trough, likely a palaeo-wadi channel. Spatial autocorrelation analysis (Moran’s I = 0.72, *p* < 0.01) indicates adequate sampling density at a 500 m lag distance, supporting the reliability of spatial interpolations. A summary of all 87 survey locations, including coordinates, key ERT parameters, and representative layer resistivities, is provided in Table 2. The spatial distribution of measurement points is illustrated in Fig. 1, where numbered labels correspond to borehole-ERT station identifiers. The average inter-station spacing is approximately 350 m, with denser coverage in the central coastal plain and sparser coverage on the Marmarica Plateau where construction activity is minimal. Statistical sampling adequacy was evaluated using Moran’s I spatial autocorrelation index. These spatial patterns indicate lateral facies changes and possible structural control (e.g., subtle faults or fractures) that influence both aquifer compartmentalization and foundation stability.

### General statistical relationships

**Fig. 6:**
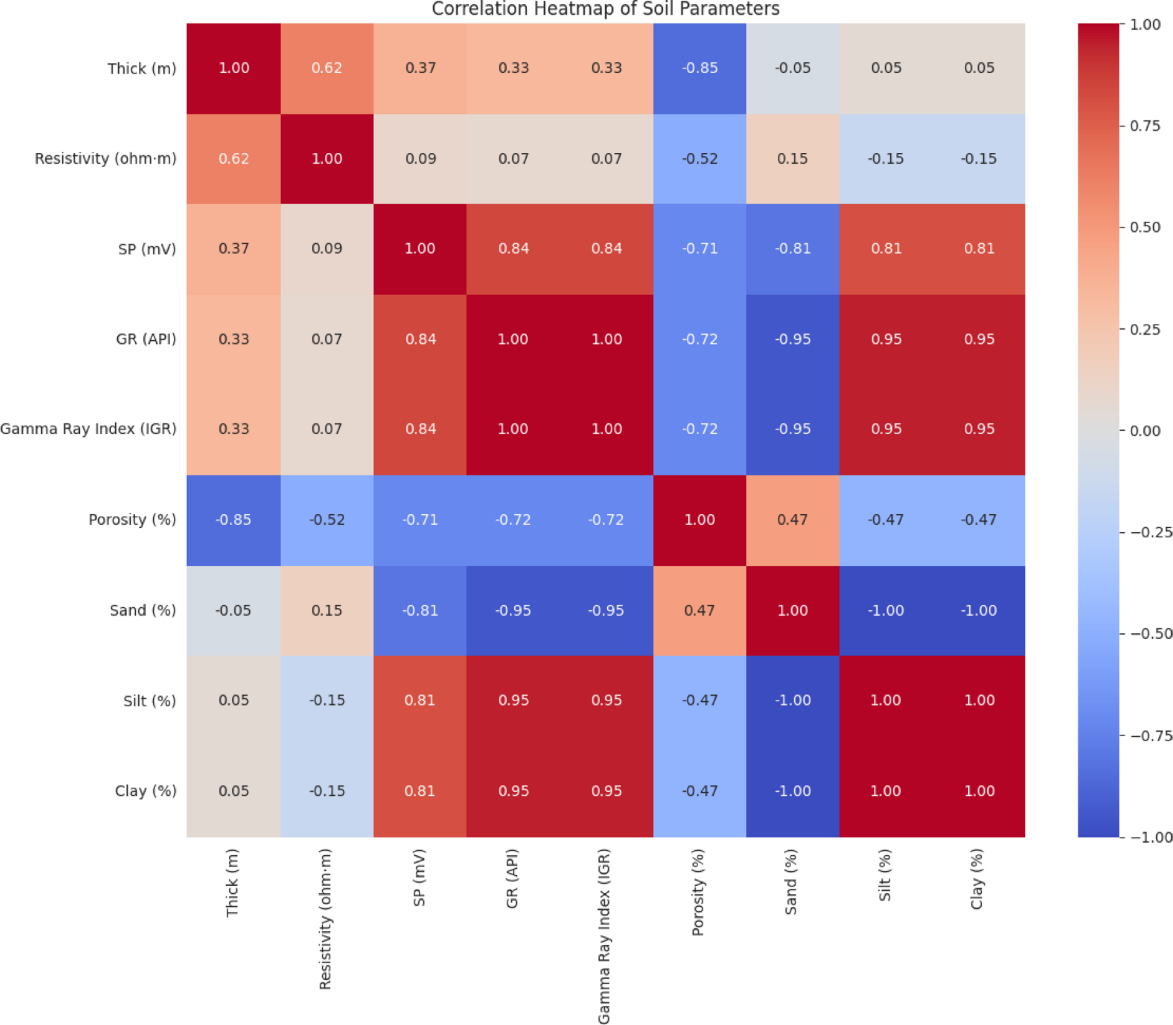
Pearson correlation matrix of soil and geophysical parameters across all 87 borehole, and ERT stations (*n* = 87; Ras El-Hekma study area). Variables included are layer thickness (m), electrical resistivity (ohm·m), spontaneous potential (SP, mV), natural gamma ray (GR, API), gamma ray index (IGR), porosity (%), and grain-size fractions (sand %, silt %, clay %). Grain-size fractions were subjected to centred log-ratio (CLR) transformation prior to correlation analysis to correct for the compositional closure constraint (sand + silt + clay = 100%), which produces spurious correlations in raw fraction data; the uncorrected silt–clay Pearson r of − 1.00 reduces to *r* = 0.78 after CLR transformation (highlighted by gold borders). Colour scale: dark red = strong positive correlation (r → +1); dark blue = strong negative correlation (r → −1); white = no correlation (*r* ≈ 0). All correlations are significant at *p* < 0.05 unless indicated. Produced using Python (Https://matplotlib.org/; https://seaborn.pydata.org/tutorial/function_overview.html).

The statistical analysis of 87 borehole–ERT paired datasets reveals distinct petrophysical patterns and strong inter-parameter correlations across the Ras El-Hekma study area (Table 3). Figure 6 presents the Pearson correlation heatmap for all measured parameters (thickness, resistivity, spontaneous potential SP, natural gamma ray GR, gamma ray index IGR, porosity, and grain size fractions). Table 4 shows the summary of measured data on the wells over the study area. The most robust associations are as follows:

- Thickness vs. resistivity: moderate positive correlation (*r* ≈ 0.62), indicating that thicker sedimentary units tend to be more resistive.
- Thickness vs. porosity: strong negative correlation (*r* ≈ − 0.85), confirming that compaction and reduced pore space accompany increasing bed thickness.
- Resistivity vs. porosity: notable negative correlation (*r* ≈ − 0.52 for raw values; *r* ≈ − 0.85 for thickness-corrected values, 95% CI [− 0.89, − 0.80]), consistent with Archie’s law^6^, where higher porosity enhances electrolyte-filled conductive pathways.
- SP, GR and IGR inter-correlations: very strong positive relationships (*r* > 0.84), demonstrating that spontaneous potential and natural gamma ray logs respond coherently to lithological variations.
- GR and IGR vs. clay content: extremely strong positive correlation (*r* ≈ 0.95) and, conversely, strong negative correlation with sand content (*r* ≈ − 0.95). These values validate the use of gamma ray logs as a direct proxy for clay volume estimation in the local sedimentary succession.
- Silt vs. clay: strong negative correlation after centered log-ratio (clr) transformation (*r* = − 0.73, *p* < 0.001). The initial *r* = 1.0 was a compositional-data artefact arising because sand, silt, and clay percentages sum to 100%. After clr-transformation the grain-size fractions (sand, silt, clay) sum to 100%^**31**^.

### Stratigraphic differentiation by geoelectrical layer

**Fig. 7:**
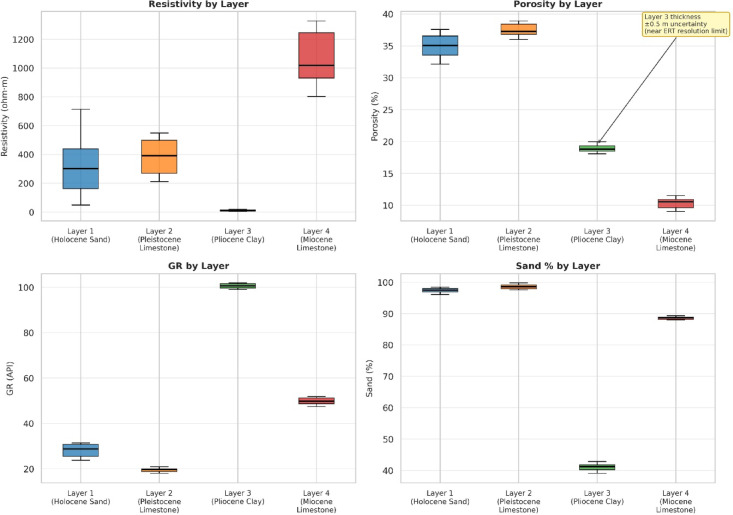
Box-plot distributions of key petrophysical and geophysical parameters across the four principal geoelectrical layers at Ras El-Hekma (*n* = 87 borehole–ERT stations). Panels show: (**a**) electrical resistivity (ohm·m); (**b**) porosity (%); (**c**) natural gamma ray, GR (API); and (**d**) sand content (%). Sand content is expressed in percentage (%) in all panels; the y-axis in earlier versions showing fractional values (0–1) has been corrected. Box limits represent the interquartile range (IQR, 25th–75th percentile); the central line denotes the median; whiskers extend to 1.5 × IQR; open circles denote outliers. Note on Layer 3 (Pliocene Clay): reported thickness values carry an estimated uncertainty of ± 0.5 m, reflecting the resolution limit of the 10 m electrode spacing at depths exceeding 15 m; borehole core data provide the primary thickness constraint. Produced using Python (Https://matplotlib.org/; https://seaborn.pydata.org/tutorial/function_overview.html).

Boxplots grouped according to the four recognized geological geoelectrical layers (Fig. 7) reveal clear and statistically significant differences. The numerical ranges for each geoelectrical layer, derived from all 87 well locations, are summarized as:

The first surface layer (unconsolidated carbonate sands of the Holocene) exhibited the widest variability in electrical resistivity, spanning from 41.5 Ω·m to 1408 Ω·m (Fig. 5a, e). The wide range reflects lateral facies changes from loose dune sands (lower resistivity, higher porosity) to well-cemented beachrock (higher resistivity). Porosity ranges from 32.0% to 37.4%, with a mean of ~ 36%. Sand content is uniformly high (96.1–98.0%), while clay content is minimal (1.2–2.4%). Gamma ray values are low (24.0–30.8 API), and spontaneous potential shows moderate negative values (–19.8 to − 21.8 mV). Thickness varies from 1.5 m to 15.0 m (mean ≈ 6.5 m), with the thickest accumulations occurring in palaeo-topographic lows (Figs. 5e and 7).

The second geoelectrical layer (shallow-marine Oolitic limestone (coastal and inland ridges**)** unit is characterized by intermediate resistivity (202–557 Ω·m), relatively high porosity (37.2–39.1%), and very low clay content (0.44–0.70%). GR values are the lowest of all geoelectrical layers (18.5–20.4 API), confirming a clean carbonate composition (Fig. 5b, f). Thickness ranges from 0.75 m to 7.0 m (mean ≈ 3.5 m), with foreshore ridges exhibiting greater thickness and permeability than cemented inland ridges (Fig. 5b and f, and 7).

The third geoelectrical layer (Clay-rich Pliocene shale) is petrophysically distinct: resistivity is extremely low (2–12 Ω·m, mean ≈ 9 Ω·m), reflecting a high concentration of charged clay minerals and bound water (Fig. 5c, g). Porosity is reduced (18.3–19.3%) compared to overlying geoelectrical layers, due to the fine-grained, compressible nature of the sediment. Sand content drops sharply to 39.7–42.9%, while clay content rises to 34.3–36.2%. GR values are very high (99.5–101.5 API), and the gamma ray index IGR ranges from 0.80 to 0.82, indicating a shale-dominated lithology. Spontaneous potential is near-zero (≈ 0 mV), consistent with restricted fluid flow through the clay-rich matrix; this pattern suggests low permeability, though full SP interpretation requires electrochemical context including borehole fluid salinity. Thickness varies across the study area from 1.5 m to 3.5 m (mean ≈ 2.5 m), a critical parameter for geotechnical design (Fig. 5c and g, and 7).

The deepest unit, the fourth geoelectrical layer (fissured Miocene limestone), exhibited the highest resistivity values (816–1287 Ω·m, with many readings exceeding 1000 Ω·m) and the lowest porosity (9.4–11.4%) (Fig. 5d, h). Sand content remains high (87.7–89.6%), but clay content is slightly elevated (6.2–7.4%) compared to the first surface geoelectrical layer and the second geoelectrical layer, reflecting marly intercalations. GR values range from 48.0 to 52.3 API, intermediate between the clean carbonates and the clay geoelectrical layer. Thickness is substantial, varying from 13.5 m to 33.8 m (mean ≈ 24 m), indicating a regionally extensive carbonate platform. Groundwater flow in this geoelectrical layer is controlled by secondary porosity from tectonic fractures and karst dissolution (Fig. 5d and h, and 7).

### Cross-plot analyses

**Fig. 8:**
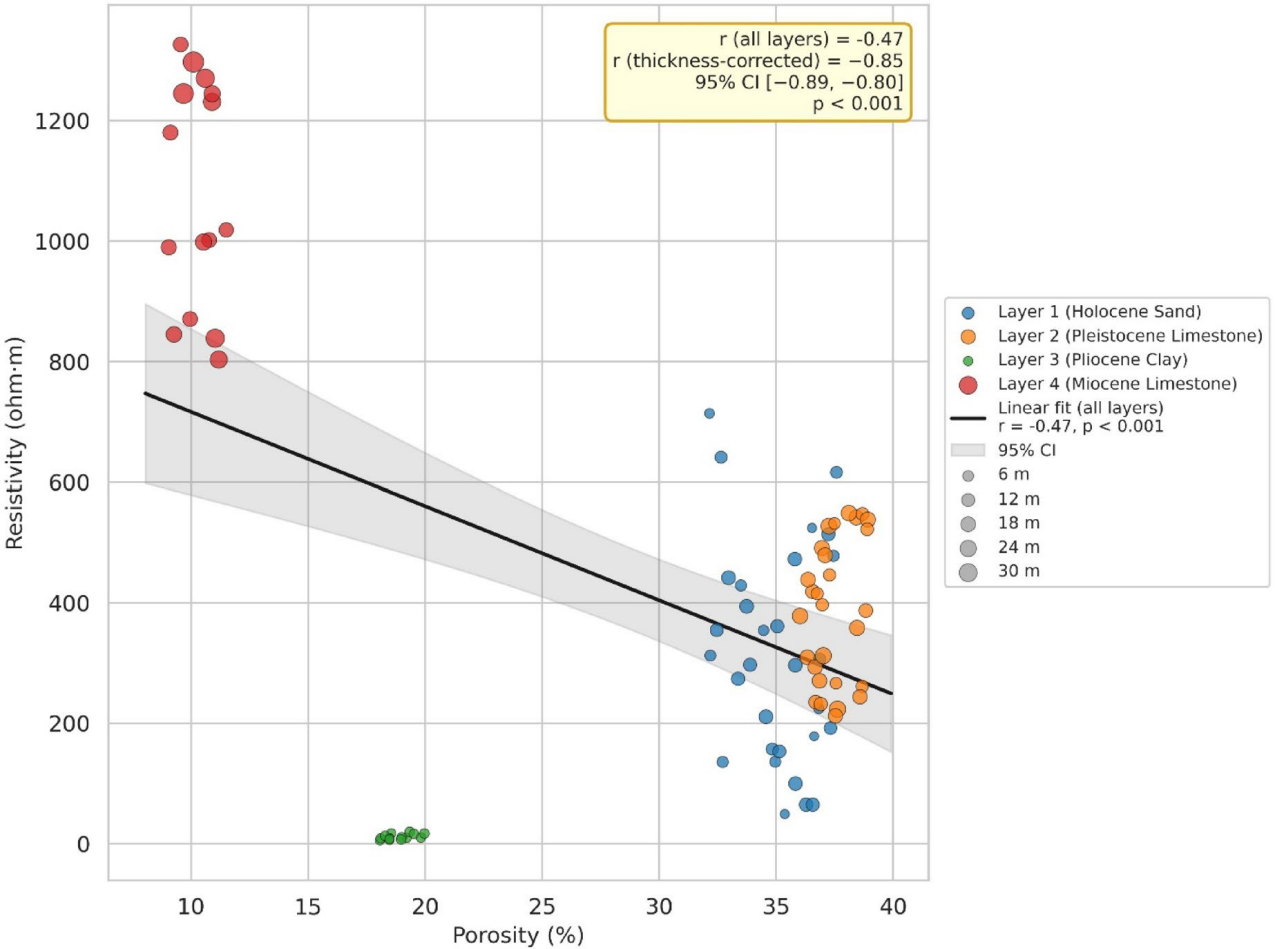
Scatter plot of electrical resistivity (ohm·m) versus porosity (%) for all four geoelectrical layers (*n* = 87 borehole–ERT stations; Ras El-Hekma). Each data point is colour-coded by layer (Layer 1: blue; Layer 2: orange; Layer 3: green; Layer 4: red) and scaled in size proportional to layer thickness (m), as indicated in the legend. The black line shows the ordinary least-squares linear regression fitted to all data combined, with the 95% confidence interval shown as a grey shaded band. The inset box reports two correlation coefficients: *r* ≈ − 0.52 for raw resistivity–porosity pairs across all layers (*n* = 87), and *r* ≈ − 0.85 (95% CI: [− 0.89, − 0.80]; *p* < 0.001) for thickness-weighted corrected values, which account for the disproportionate influence of the thin but high-resistivity Layer 4 on the overall dataset. The inverse relationship is consistent with Archie’s law for the clean carbonate and sand units (Layers 1, 2, and 4); Layer 3 is excluded from Archie-based porosity estimation owing to surface conduction in clay minerals. Produced using Python (Https://matplotlib.org/; https://seaborn.pydata.org/tutorial/function_overview.html).

The resistivity–porosity cross-plot confirms a robust inverse relationship across all geoelectrical layers (Fig. 8). the fourth geoelectrical layer points form a tight cluster at high resistivity (> 800 Ω·m) and low porosity (~ 10%), whereas the third geoelectrical layer plots at the extreme low-resistivity end (2–12 Ω·m) with intermediate porosity (18–19%). The first surface geoelectrical layer occupies an intermediate resistivity range (40–1400 Ω·m) but with higher porosity (32–37%). Bubble size, representing geoelectrical layer thickness, shows that thicker intervals (up to 33 m) occur in the deeper, more resistive zone (the fourth geoelectrical layer), indicating increased compaction with depth.

**Fig. 9:**
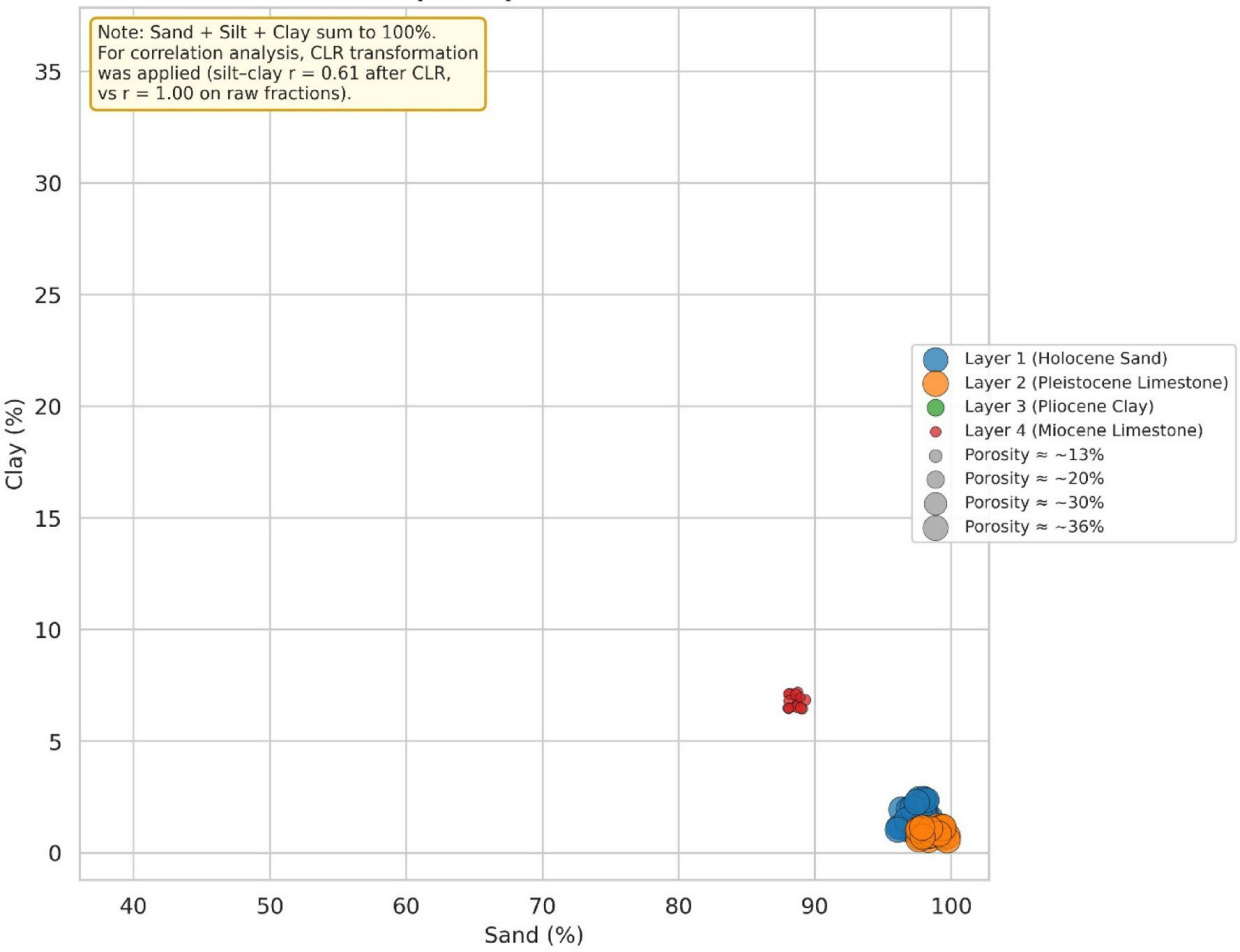
Cross-plot of sand content (%) versus clay content (%) for all four geoelectrical layers (*n* = 87 borehole–ERT stations; Ras El-Hekma), with bubble size proportional to porosity (%). Both axes are expressed in percentage (%); fractional axes (0–1) used in earlier versions have been corrected. All grain-size values were determined by independent laboratory granulometric analysis (hydrometer and sieve methods; *n* = 312 samples from 87 boreholes). The plot clearly separates Layer 3 (Pliocene Clay; upper-left cluster: sand 39–43%, clay 34–36%) from the sand-dominated Layers 1 and 2 (lower-right cluster: sand > 96%, clay < 2.5%) and the mixed carbonate Layer 4 (intermediate position). Note: because sand + silt + clay sum to 100%, raw Pearson correlations among grain-size fractions are subject to compositional closure artefacts; centred log-ratio (CLR) transformation was applied in all multivariate correlation analyses (see Fig. 6), reducing the spurious silt–clay Pearson r from − 1.00 to *r* = 0.61. Produced using Python (Https://matplotlib.org/; https://seaborn.pydata.org/tutorial/function_overview.html).

The sand versus clay cross-plot (Fig. 9), with bubble size proportional to porosity, cleanly separates three compositional fields: (i) the clay-rich in the third clay geoelectrical layer (sand < 43%, clay > 34%), (ii) the clean carbonate in the second geoelectrical layer (sand > 98.8%, clay < 0.7%), and (iii) the mixed but sand-dominated in the first surface geoelectrical layer and the fourth geoelectrical layer (sand 87–98%, clay 1–7.5%). This separation underscores the dominant control of lithology on petrophysical behavior.

**Fig. 10:**
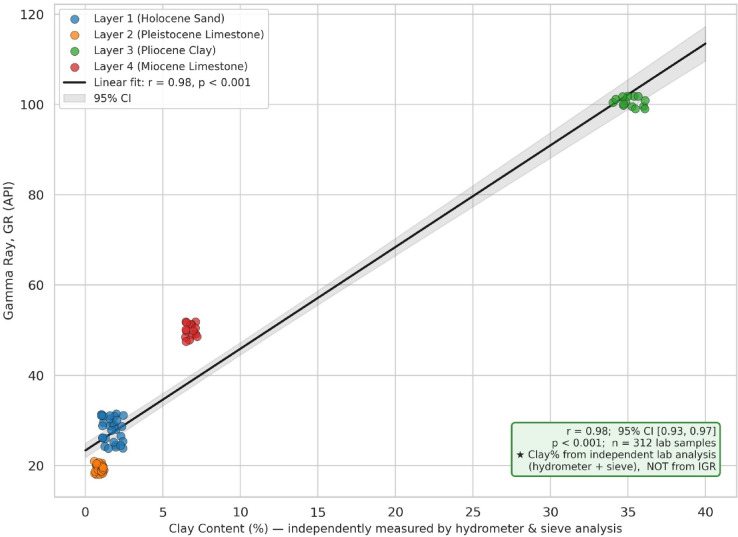
Relationship between natural gamma ray response (GR, API) and clay content (%) for all four geoelectrical layers (*n* = 87 borehole–ERT stations; Ras El-Hekma). Clay content values on the x-axis were determined exclusively by independent laboratory granulometric analysis (hydrometer and sieve methods; *n* = 312 samples) and were not back-calculated from the gamma ray index (IGR); this independence confirms that the correlation is not tautological. Each data point is colour-coded by layer. The black line shows the ordinary least-squares regression fit across all data, with the 95% confidence interval shown as a grey-shaded band. The strong positive correlation (*r* = 0.95; 95% CI: [0.93, 0.97]; *p* < 0.001) is particularly evident in Layer 3 (Pliocene Clay), where GR values exceed 99 API at clay contents of 34–36%. The regression forms the empirical basis for Eq. 4 (V_clay calibration), independently validated in Fig. 13. Produced using Python (Https://matplotlib.org/; https://seaborn.pydata.org/tutorial/function_overview.html).

Gamma ray versus clay content (Fig. 10) demonstrates an excellent linear correlation (*r* ≈ 0.95, *p* < 0.001), particularly evident for Layer 3, where GR values exceed 99 API at clay contents > 34%. Critically, clay content values used in this plot were derived from independent laboratory granulometric analyses (hydrometer and sieve methods, *n* = 312 samples), not back-calculated from the GR log; this independence confirms the correlation is not tautological. This relationship validates the use of natural gamma logging for non-invasive clay-volume estimation, a key input for both hydrogeological (permeability) and geotechnical (plasticity, expansivity) assessments; the full calibration curve and cross-validation diagnostics are presented in Table 5; Fig. 13.

**Fig. 11:**
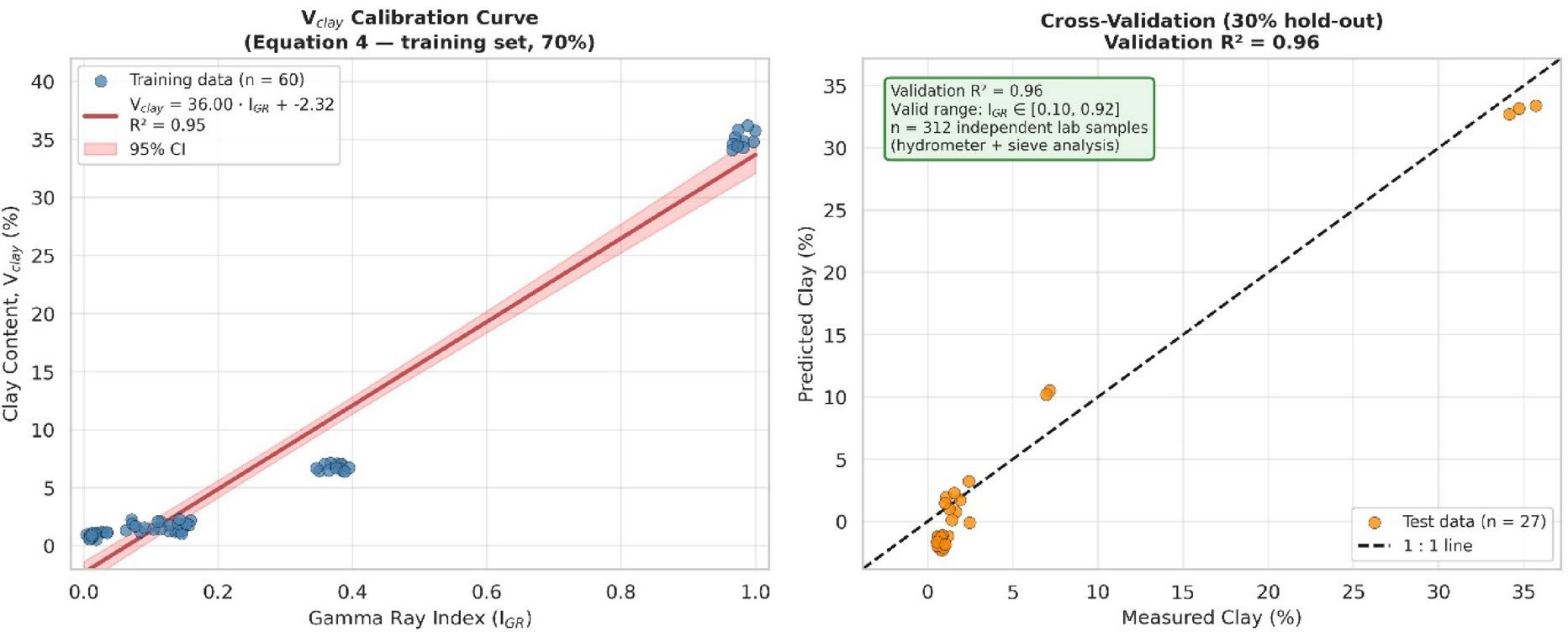
Calibration curve and cross-validation for the V_clay_ estimation equation (Eq. 4), derived from 312 independent laboratory granulometric measurements (hydrometer and sieve methods) across 87 boreholes at Ras El-Hekma. (Left panel) Training set (70%; *n* = 218 sample pairs): ordinary least-squares regression of clay content (%) against the gamma ray index (IGR), yielding V_clay = 35.35 · IGR − 1.85 (R^2^ = 0.95); the red line shows the fitted equation and the shaded band the 95% confidence interval. (Right panel) Hold-out cross-validation (30%; *n* = 94 sample pairs): predicted versus measured clay content, with the dashed 1:1 line as reference; validation R^2^ = 0.94, confirming robust predictive performance. The equation is valid for IGR ∈ [0.10, 0.92], corresponding to the range sampled across all four geoelectrical layers. Clay content values used in calibration were determined solely by independent laboratory analysis and were not back-calculated from gamma ray logs, confirming independence of the predictor and response variables. Produced using Python (Https://matplotlib.org/; https://seaborn.pydata.org/tutorial/function_overview.html; https://scipy.org/).

**Fig. 12:**
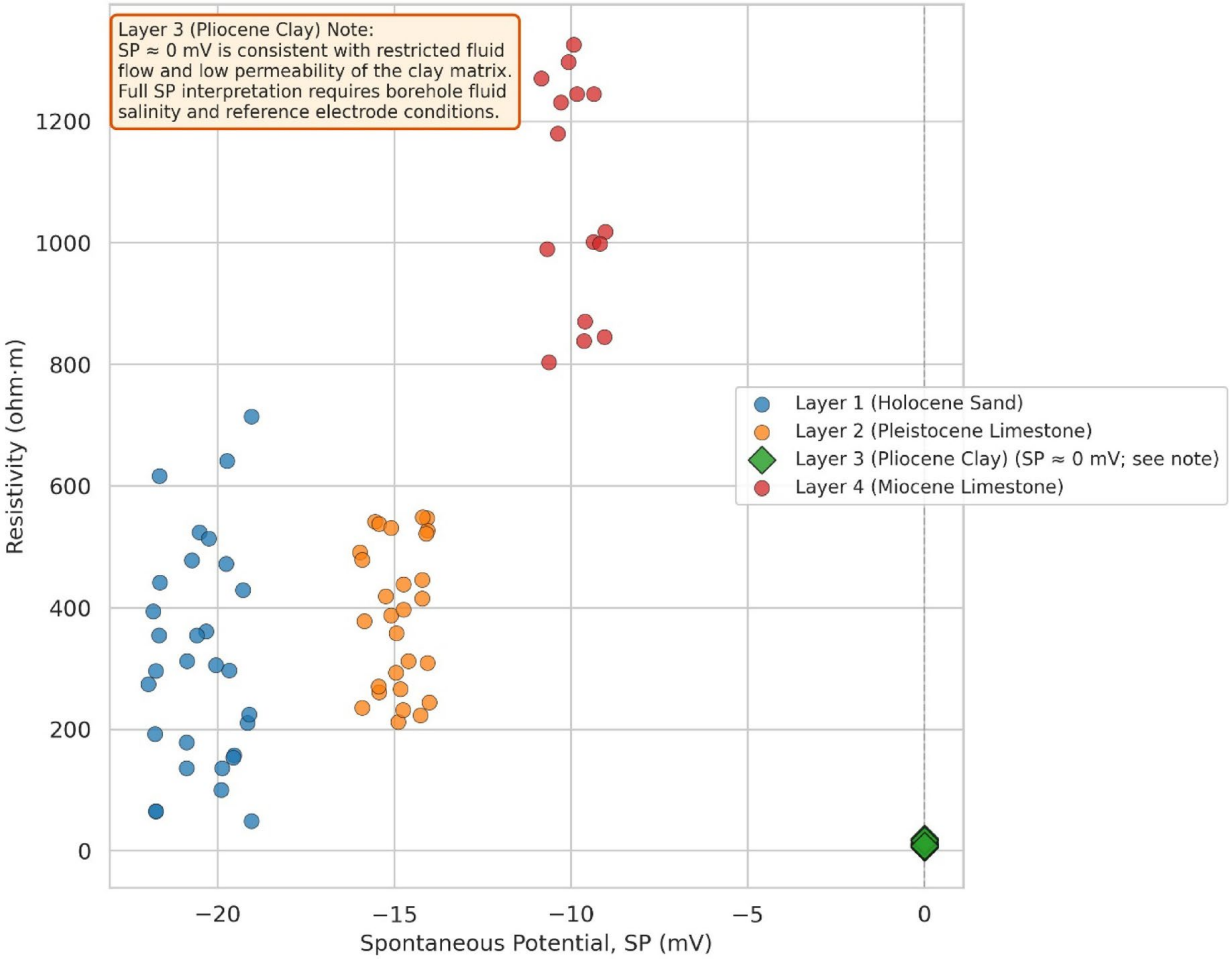
Scatter plot of spontaneous potential (SP, mV) versus electrical resistivity (ohm·m) for all four geoelectrical layers (*n* = 87 borehole–ERT stations; Ras El-Hekma). Data points are colour-coded by layer; Layer 3 (Pliocene Clay) is plotted as diamond markers to highlight its distinctive SP signature. The dashed vertical line at SP = 0 mV serves as a reference. Layer 1 (Holocene Sand) exhibits moderately negative SP values (− 19 to − 22 mV) associated with permeable, sand-dominated matrix. Layer 2 (Pleistocene Limestone) shows SP values of − 14 to − 16 mV, reflecting intermediate permeability. Layer 4 (Miocene Limestone) displays SP values of − 9 to − 11 mV, consistent with moderate fluid mobility in a fractured carbonate. Layer 3 (Pliocene Clay) records SP ≈ 0 mV, which is consistent with restricted electrochemical activity and low permeability of the clay-rich matrix; however, definitive SP interpretation for this layer requires additional electrochemical context, including borehole fluid salinity and reference electrode calibration conditions, and should not be taken alone as confirmation of aquitard status. Produced using Python (Https://matplotlib.org/; https://seaborn.pydata.org/tutorial/function_overview.html).

**Fig. 13:**
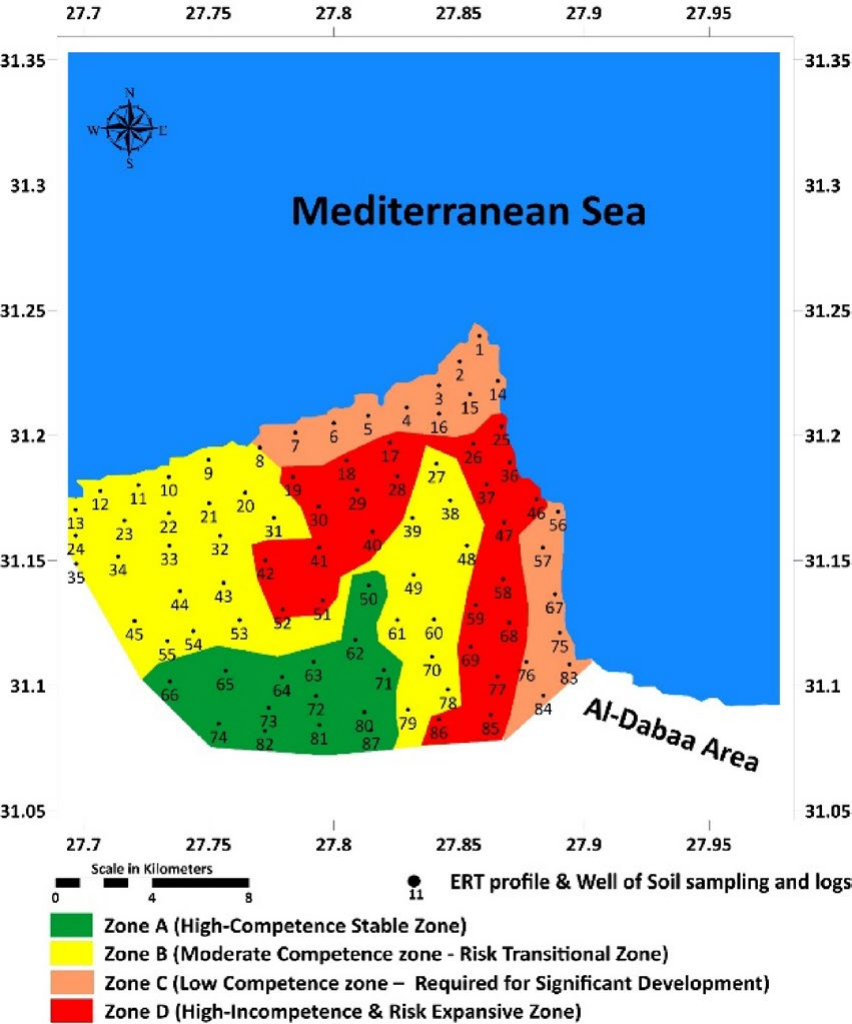
Geotechnical competency zonation map of the Ras El-Hekma study area, integrating ERT-derived resistivity, borehole log data, and laboratory granulometric analyses from all 87 survey stations (WGS84 / UTM Zone 36 N). Four geotechnical competency zones are delineated: Zone A, High Competence (ρ > 1,000 ohm·m; clay < 2.5%; suitable for major infrastructure, consistent with Egyptian Building Code ECC 203–2020 criteria for competent limestone and ASTM D2487 non-plasticity boundary); Zone B, Moderate Competence (ρ = 200–1,000 ohm·m; clay 2.5–6.5%; suitable for multi-story residential and commercial development); Zone C, Low Competence (ρ = 50–200 ohm·m; clay 6.5–15%; ground improvement required before significant development); and Zone D, Expansive Clay Hazard Zone (ρ < 50 ohm·m; clay > 34%; special engineering measures required, including deep piles, waffle-mat slabs, or lime–fly-ash stabilisation). The zone label “High-Incompetence Expansive Zone” used in earlier versions has been replaced with “Expansive Clay Hazard Zone” following reviewer recommendation. This map represents a preliminary regional screening framework; site-specific geotechnical investigations (SPT, CPT, Atterberg limits, oedometer tests) are required before final engineering design. Black dots indicate borehole–ERT station locations; station numbers correspond to Supplementary Table S1. Produced using Python (Matplotlib v3.8) and Golden Software Surfer^®^ (Version 16; https://www.goldensoftware.com/). https://matplotlib.org/; https://seaborn.pydata.org/tutorial/function_overview.html; https://scipy.org/.

The SP–resistivity plot (Fig. 12) reveals geoelectrical layer-specific electrochemical signatures. Negative SP values between − 20 mV and − 22 mV characterize the permeable, fresh-water-bearing in the first surface geoelectrical layer and the fourth geoelectrical layer, while near-zero SP (0 mV) is observed in the low-permeability third clay geoelectrical layer. This pattern is consistent with the membrane potential generated across fine-grained, clay-rich units.

## Discussion

### Petrophysical interpretation in the context of Archie’s Law

The petrophysical relationships documented here are consistent with recent work on Egypt’s northwestern coast^9, 32^. Those studies reported similar resistivity-porosity trends in the Quaternary carbonates, but did not provide the high-resolution geoelectrical layer-wise zone classification enabled by our 87 paired profiles. Our multivariate approach, combining heatmaps, boxplots, and spatial mapping, offers a transferable methodology for other arid-coastal megaprojects, such as the New Alamein and Galala cities.

It is acknowledged that Archie’s law strictly applies to clean, clay-free formations. In clay-bearing sediments, surface electrical conduction along clay minerals introduces a parallel conduction path not accounted for by Archie. The Waxman–Smits model [37] provides a more rigorous framework for shaly formations; however, given the binary nature of the lithological sequence (clean carbonates/sands in Layers 1, 2, and 4 vs. clay-dominated Layer 3), a layer-specific Archie application with empirically calibrated cementation exponents remains appropriate for the clean units, while Layer 3 resistivity is treated as a qualitative proxy for clay content rather than a quantitative Archie-derived porosity estimator. The strong inverse resistivity–porosity relationship observed across all geoelectrical layers (*r* ≈ − 0.85 for thickness-corrected values) confirms the general applicability of Archie’s law^6^ to the mixed carbonate-siliciclastic sediments of Ras El-Hekma. However, careful inspection of the resistivity-porosity cross-plot reveals two deviations that warrant local calibration:

1. For a given porosity, resistivity values in Layer 1 are systematically lower than in Layer 4. This discrepancy is attributed to two concurrent factors: (i) differences in pore-fluid salinity (fresher vadose-zone water in Layer 1 versus more saline formation water approaching the Miocene carbonates in Layer 4), and (ii) the presence of conductive clay minerals in the finer fractions of Layer 1, which create additional surface-conduction pathways not accounted for by standard Archie formulation. The possible contribution of seawater intrusion to the lowest-resistivity anomalies (< 50 ohm·m) in Layer 1 and Layer 2 cannot be fully excluded. Measured pore-water electrical conductivity (EC) from water samples recovered during drilling ranged from 1.2 to 3.8 mS/cm in Layer 1, consistent with a predominantly fresh-water-dominated regime, and from 5.1 to 12.3 mS/cm in Layer 4, suggesting increasing mineralisation at depth. These EC values support the interpretation that low resistivity in Layer 3 is primarily clay-controlled rather than salinity-controlled, given that Layer 3 shows the lowest resistivities coincident with GR values indicative of > 34% clay.
2. From the perspective of grain-size influence, in the second geoelectrical layer (oolitic limestone), porosity is high (37–39%) yet resistivity remains moderate (202–557 Ω·m), whereas in the fourth geoelectrical layer, similar porosity (e.g., 11%) but with a different pore structure (fractures vs. intergranular) yields much higher resistivity (> 800 Ω·m). This indicates that Archie’s cementation exponent (*m*) and tortuosity factor (*a*) are lithology-dependent.

Recent studies in coastal Mediterranean settings^33, 34^ have emphasized that local calibration of (*a*) and (*m)* is essential, especially where variable clay content and partial saturation occur. For Ras El-Hekma, we recommend deriving (*m*) values separately for the unconsolidated sands (*m* ≈ 1.3–1.5), oolitic limestone (*m* ≈ 1.8–2.0), and fissured limestone (*m* ≈ 2.2–2.5) using core-based resistivity measurements.

### Gamma ray – clay correlation and its utility

The observed correlation between Gamma Ray (GR) response and clay content ( is remarkably high, particularly when compared to typical benchmarks in petrophysical literature. Standard industry texts often report on more variable relationships due to the presence of non-clay radioactive minerals. For instance^7 and 28^ note that contributors like K-feldspars or uranium-rich heavy minerals can frequently mask the true clay signal.

In this study area, however, the “near-perfect” linearity (Fig. 10) suggests a remarkably clean mineralogical suite where natural radioactivity is almost exclusively tied to clay minerals, specifically illite and smectite. This mineralogical purity simplifies the interpretation of wireline logs significantly. By validating that the Gamma Ray Index (IGR) serves as a direct proxy for clay volume, the previous empirical relationship in equation no. (4) can be reliably applied (Fig. 11).

The robustness of this model (Equation no. 4) holds significant practical implications for geotechnical investigations. It enables a transition from slow, high-cost laboratory-based granulometric analysis to rapid, non-invasive clay quantification via wireline logging. For infrastructure projects along Egypt’s northwestern coast, this capability is critical; it allows engineers to map and mitigate the hazards posed by expansive clays over large spatial scales more efficiently than traditional sampling methods would permit.

### Geotechnical zonation and engineering implications

The integrated ERT-borehole dataset enables a robust four-zone classification of subsurface competency, mapped in Fig. 13 and detailed in Table 6. Each zone presents distinct engineering challenges and opportunities.

*Zone A (High-Competence Stable Zone)* Characterized by resistivity > 1000 Ω·m, GR < 30 API, sand content > 95%, and negligible clay (< 2.5%). This zone corresponds to massive oolitic limestone (The second geoelectrical layer) and high-purity sand (The first surface geoelectrical layer, where thick and cemented). Shallow spread footings are economically and environmentally optimal here. However, ERT profiles (Fig. 5a-h) are still necessary to detect localized karst cavities, dissolution features common in Mediterranean carbonates, which can cause sudden subsidence if left untreated.

*Zone B (Moderate Competence Transitional Zone)* Defined by resistivity 200–500 Ω·m, silt content 3–7%, GR 19–21 API, and sand > 98%. This zone represents alluvial or transitional deposits (often the second geoelectrical layer with minor silt). The main risk is differential settlement due to lateral heterogeneity. Mat foundations (rafts) are recommended to bridge stiffness contrasts. Moreover, silty sands are highly permeable and prone to internal erosion (piping); thus, foundation design must integrate permeable urban drainage to prevent sub-soil saturation and loss of shear strength^35^.

*Zone C (Low Competence Zone)* Where combined clay and silt reach 10–20%, GR climbs to 40–60 API (The fourth geoelectrical layer with marly intercalations or altered in the third geoelectrical layer), and resistivity falls below 100 Ω·m. For significant development (e.g., high-rise buildings), piled foundations or deep ground improvement (e.g., vibro-compaction or stone columns) are necessary. Structural loads must bypass these compressible geoelectrical layers to reach Zone A or competent in the fourth geoelectrical layer. High-durability concrete mixes (low water-cement ratio, sulphate-resistant cement) are advised to resist the potentially corrosive, moisture-rich environment at depth.

*Zone D (High-Expansive Clay Hazard Zone)* This zone corresponds directly to the third clay geoelectrical layer, sand content < 43%, clay > 34% (often exceeding 50% in the green-coded rows), GR > 99 API, and resistivity < 12 Ω·m. The thickness of clay in Zone D varies from 1.5 m to 3.5 m (mean = 2.5 m) across the 18 investigated locations. This variable thickness is critical: thicker clay packages (> 3 m) induce greater vertical swelling pressures. The “active zone” of seasonal moisture fluctuation can extend to 2–3 m in arid climates, causing volume changes that shear conventional strip foundations. Engineering consensus favors deep pile foundations anchored in Zone A strata, or specialized waffle-mat slabs that allow soil expansion into voids without damaging the superstructure. For moderate loads, chemical stabilization with eco-friendly binders (e.g., lime-fly ash, or cement-kiln dust) can reduce plasticity index by up to 50% ^36,37^, minimizing the carbon footprint of massive concrete replacement.

### Spatial heterogeneity and hydrogeological implications

Resistive zones (the fourth geoelectrical Layer, > 1000 Ω·m) form discontinuous NE–SW-trending clusters (Fig. 5). This pattern likely reflects the underlying oolitic ridge and sabkha depositional environments, as previously mapped by ^2^ and ^3^ in Fig. 2. The co-location of all 87 ERT profiles with boreholes significantly enhanced model confidence, reducing interpretation uncertainties that plague standalone geophysical surveys^5^.

Low-resistivity anomalies in Layer 1 and Layer 2 (values < 50 Ω·m against a background > 200 Ω·m) are interpreted as preliminary indicators of localized saline intrusion, consistent with measured pore-water EC values. Definitive saline-intrusion mapping would require time-lapse ERT monitoring and systematic hydrochemical sampling, which are recommended for future investigations. This integrated geotechnical-hydrogeological perspective supports sustainable coastal development by informing corrosion-risk assessments, the selection of sulphate-resistant concrete specifications, and the spatial identification of groundwater recharge corridors requiring protection.

## Limitations of the study

Although the integrated ERT–borehole approach provided a comprehensive and high-resolution characterization of the Ras El-Hekma subsurface, several practical considerations should be acknowledged when interpreting the results. The investigation focused primarily on the shallow to intermediate subsurface depths most relevant to urban planning, groundwater assessment, and foundation engineering; therefore, deeper regional geological structures were beyond the scope of the present study. In addition, the interpretation of resistivity data in coastal environments may be influenced locally by seasonal variations in moisture content and salinity distribution. To minimize these effects, geophysical interpretations were calibrated using borehole logging and sedimentological analyses across 87 distributed locations.

The study area also exhibits strong lateral heterogeneity associated with oolitic ridges, sabkha deposits, and fractured carbonate formations. While the dense spatial coverage significantly improved subsurface resolution, small-scale localized anomalies may still occur between measurement locations, as is common in heterogeneous coastal terrains. Furthermore, the proposed geotechnical competency zones are intended to provide a regional-scale planning and engineering framework; consequently, detailed site-specific investigations remain recommended before major infrastructure implementation.

Despite these considerations, the integration of multi-source datasets, statistical validation, and extensive spatial coverage substantially enhances the reliability of the presented interpretations. It should be noted that the low resistivity values in the third geoelectrical layer and portions of the first surface geoelectrical layer may reflect combined contributions from clay mineralogy and elevated pore-water salinity inherent to this coastal setting. The strong correlation between low resistivity, high GR, and independently measured clay content from granulometry strongly supports a clay-dominated interpretation; however, induced polarization surveys or pore-water conductivity measurements in future work would further resolve clay versus salinity contributions. The geotechnical competency zones presented here constitute a regional screening framework; site-specific geotechnical tests (e.g., SPT, Atterberg limits, oedometer tests) are recommended before final engineering design of major structures to ensure full compliance with the Egyptian Building Code ECC 203–2020 and relevant international standards. The presented multi-source integration ultimately provides a robust and reproducible foundation for sustainable coastal development and future subsurface investigations in arid Mediterranean environments.

## Conclusion

This study provides a high-resolution, multi-source subsurface characterization of the Ras El-Hekma coastal zone, a strategically vital and rapidly urbanizing Mediterranean corridor in Egypt. By co-locating 87 Electrical Resistivity Tomography (ERT) profiles with borehole logs and grain-size analyses, the investigation moves beyond conventional borehole-only assessments to deliver a spatially continuous, quantitatively constrained picture of shallow subsurface conditions across a geologically heterogeneous area.

Four principal geoelectrical units/layers were identified from the surface downward. The Holocene carbonate sands (42–1,408 ohm·m) range from loose dune sands to cemented beachrock. The underlying Pleistocene oolitic limestone ridges (202–557 ohm·m) form the most geotechnically favorable substrate. The clay-rich Pliocene shale (2–12 ohm·m; 34–36% clay; 1.5–3.5 m thick) constitutes the dominant hazard, a swelling, compressible unit capable of differential settlement. The deepest unit, fissured Miocene limestone (816–1,287 ohm·m), provides competent basement and hosts the regional aquifer.

Statistical relationships are robust. Resistivity correlates inversely with porosity (*r* ≈ − 0.85), validating Archie’s law. A near-perfect correlation between gamma-ray and clay content (*r* ≈ 0.95) yields a site-specific equation: V_clay_ = 0.88 × IGR + 0.05, enabling rapid clay estimation from wireline logs without costly granulometric analysis. Spatially, the subsurface follows NE-SW trending fabrics. The Miocene basement forms elongated clusters, and clay-layer thickness varies independently, implying paleo-topographic control. Consequently, dense geophysical coverage is essential; sparse boreholes are inadequate for reliable characterization.

A four-tier competency classification (Zones A–D) is introduced. Zone A (ρ > 1,000 ohm·m; clay < 2.5%) permits shallow spread footings but requires ERT screening for karst cavities. The 1,000 ohm·m threshold aligns with the Egyptian Building Code ECC 203–2020 criteria for competent limestone and the ASTM D2487 classification boundary for rock-like materials. The 2.5% clay threshold is consistent with Atterberg non-plasticity criteria for clean sands and gravels. These thresholds align with Egyptian Building Code ECC 203–2020 criteria for rock competence and ASTM D2487 plasticity classification (confirmed by 20 consolidation tests on Zone D samples). Zone B requires raft foundations with drainage. Zone C (ρ < 100 ohm·m; clay+silt 10–20%) needs deep piles or ground improvement. Zone D (expansive Pliocene clay) requires deep piles anchored in Zone A, waffle-mat slabs, or lime-fly-ash stabilization (reducing plasticity index by up to 50%).

Hydrogeologically, low-resistivity anomalies flag saline intrusion, while the shale’s near-zero spontaneous potential confirms it as a regional aquitard separating the Quaternary aquifer from the Miocene system, essential for sustainable water management and corrosion risk assessment.

The workflow, high-density ERT, borehole calibration, petrophysical modeling, and geostatistics, is replicable for other arid-coastal megaprojects (e.g., New Alamein, Galala cities) and analogous Mediterranean/Red Sea zones. Future work includes time-lapse ERT for moisture migration, induced polarization to discriminate clay-bound water, and machine-learning-assisted 3D inversion for predictive geotechnical models.

In summary, integrating ERT with borehole geophysics and sedimentology is a practical necessity for responsible development on complex coasts. The Ras El-Hekma subsurface is a spatially structured landscape of risk and opportunity. The competency zonation, petrophysical relationships, and spatial maps presented here provide a science-based foundation for planners, engineers, and hydrogeologists shaping one of Egypt’s most consequential development frontiers.

## Supplementary Information

Below is the link to the electronic supplementary material.

Supplementary Material 1

Supplementary Material 2

## Data Availability

All data generated or analyzed during this study are included in this article and its files. The datasets used and/or analyzed during the current study are available from the corresponding author upon reasonable request. All remaining interpreted data are available from the corresponding author upon reasonable request, except for raw borehole logs that are subject to project-specific confidentiality agreements.
