# Supplementary material for: Integrated ERT, petrophysics and borehole logging for geotechnical zonation and sustainable coastal development in Ras El-Hekma, Egypt
Source: Sci Rep. 2026 Jul 14;16:22035. doi: 10.1038/s41598-026-60990-0 (PMC13369940; doi:10.1038/s41598-026-60990-0)
Supplement: Supplementary file 2 — Supplementary Material 2 [file 41598_2026_60990_MOESM2_ESM.docx]

All Calculated Electrical Parameters of Soil Samples

Fully Data includes 279 records across multiple layers.

| Well | Long | Lat | Thick (m) | Resistivity (ohm·m) | SP (mV) | GR (API) | Gamma Ray Index (IGR) | Porosity (%) | Sand (%) | Silt (%) | Clay (%) | Layer order | Layer Description |
| --- | --- | --- | --- | --- | --- | --- | --- | --- | --- | --- | --- | --- | --- |
| 1 | 27.858202000427 | 31.239767938638 | 4.5 | 44.5 | -20.2 | 29.3 | 0.1361904761904762 | 36.2 | 0.9653021470031571 | 0.01387914119873717 | 0.020818711798105752 | Layer 1 | The first surface layer (Unconsolidated carbonate sands (dune and beach), alluvial wadi fill, and evaporitic sabkha deposits.) Holocene |
| 2 | 27.850363711235 | 31.229669385064 | 5.0 | 45.0 | -20.3 | 29.0 | 0.13333333333333333 | 36.0 | 0.9661614325251788 | 0.013535426989928451 | 0.020303140484892675 | Layer 1 | The first surface layer (Unconsolidated carbonate sands (dune and beach), alluvial wadi fill, and evaporitic sabkha deposits.) Holocene |
| 3 | 27.842019725965 | 31.220075759169 | 3.0 | 43.0 | -20.0 | 30.0 | 0.14285714285714285 | 36.8 | 0.9632724908960749 | 0.014691003641570034 | 0.022036505462355048 | Layer 1 | The first surface layer (Unconsolidated carbonate sands (dune and beach), alluvial wadi fill, and evaporitic sabkha deposits.) Holocene |
| 4 | 27.829124476002 | 31.211239524792 | 3.5 | 43.5 | -20.1 | 29.8 | 0.14095238095238097 | 36.6 | 0.9638559379999075 | 0.01445762480003699 | 0.021686437200055482 | Layer 1 | The first surface layer (Unconsolidated carbonate sands (dune and beach), alluvial wadi fill, and evaporitic sabkha deposits.) Holocene |
| 5 | 27.813700745655 | 31.20795749488 | 3.75 | 43.75 | -20.1 | 29.6 | 0.13904761904761906 | 36.5 | 0.9644365418931404 | 0.014225383242743847 | 0.02133807486411577 | Layer 1 | The first surface layer (Unconsolidated carbonate sands (dune and beach), alluvial wadi fill, and evaporitic sabkha deposits.) Holocene |
| 6 | 27.800046951578 | 31.204927928808 | 5.0 | 45.0 | -20.3 | 29.0 | 0.13333333333333333 | 36.0 | 0.9661614325251788 | 0.013535426989928451 | 0.020303140484892675 | Layer 1 | The first surface layer (Unconsolidated carbonate sands (dune and beach), alluvial wadi fill, and evaporitic sabkha deposits.) Holocene |
| 7 | 27.784623221231 | 31.201140971218 | 2.75 | 42.75 | -20.0 | 30.1 | 0.14380952380952383 | 36.9 | 0.9629796967918526 | 0.014808121283258964 | 0.022212181924888445 | Layer 1 | The first surface layer (Unconsolidated carbonate sands (dune and beach), alluvial wadi fill, and evaporitic sabkha deposits.) Holocene |
| 8 | 27.770463731076 | 31.195081839073 | 4.5 | 44.5 | -20.2 | 29.3 | 0.1361904761904762 | 36.2 | 0.9653021470031571 | 0.01387914119873717 | 0.020818711798105752 | Layer 1 | The first surface layer (Unconsolidated carbonate sands (dune and beach), alluvial wadi fill, and evaporitic sabkha deposits.) Holocene |
| 9 | 27.749983039959 | 31.190285026126 | 3.75 | 43.75 | -20.1 | 29.6 | 0.13904761904761906 | 36.5 | 0.9644365418931404 | 0.014225383242743847 | 0.02133807486411577 | Layer 1 | The first surface layer (Unconsolidated carbonate sands (dune and beach), alluvial wadi fill, and evaporitic sabkha deposits.) Holocene |
| 10 | 27.734053613535 | 31.183468502463 | 4.0 | 44.0 | -20.2 | 29.5 | 0.1380952380952381 | 36.4 | 0.9647257819682108 | 0.014109687212715686 | 0.02116453081907353 | Layer 1 | The first surface layer (Unconsolidated carbonate sands (dune and beach), alluvial wadi fill, and evaporitic sabkha deposits.) Holocene |
| 11 | 27.721916907688 | 31.180186472552 | 4.0 | 44.0 | -20.2 | 29.5 | 0.1380952380952381 | 36.4 | 0.9647257819682108 | 0.014109687212715686 | 0.02116453081907353 | Layer 1 | The first surface layer (Unconsolidated carbonate sands (dune and beach), alluvial wadi fill, and evaporitic sabkha deposits.) Holocene |
| 12 | 27.70674602538 | 31.177914297998 | 1.5 | 41.5 | -19.8 | 30.8 | 0.15047619047619049 | 37.4 | 0.9609099908866242 | 0.015636003645350317 | 0.023454005468025473 | Layer 1 | The first surface layer (Unconsolidated carbonate sands (dune and beach), alluvial wadi fill, and evaporitic sabkha deposits.) Holocene |
| 13 | 27.696884951879 | 31.170340382817 | 3.0 | 43.0 | -20.0 | 30.0 | 0.14285714285714285 | 36.8 | 0.9632724908960749 | 0.014691003641570034 | 0.022036505462355048 | Layer 1 | The first surface layer (Unconsolidated carbonate sands (dune and beach), alluvial wadi fill, and evaporitic sabkha deposits.) Holocene |
| 14 | 27.865534593543 | 31.221843006044 | 2.0 | 42.0 | -19.9 | 30.5 | 0.14761904761904762 | 37.2 | 0.9618013425514456 | 0.015279462979421786 | 0.022919194469132678 | Layer 1 | The first surface layer (Unconsolidated carbonate sands (dune and beach), alluvial wadi fill, and evaporitic sabkha deposits.) Holocene |
| 15 | 27.85440927985 | 31.216541265418 | 3.0 | 93.0 | -20.0 | 30.0 | 0.14285714285714285 | 36.8 | 0.9632724908960749 | 0.014691003641570034 | 0.022036505462355048 | Layer 1 | The first surface layer (Unconsolidated carbonate sands (dune and beach), alluvial wadi fill, and evaporitic sabkha deposits.) Holocene |
| 16 | 27.842019725965 | 31.208714886398 | 3.0 | 93.0 | -20.0 | 30.0 | 0.14285714285714285 | 36.8 | 0.9632724908960749 | 0.014691003641570034 | 0.022036505462355048 | Layer 1 | The first surface layer (Unconsolidated carbonate sands (dune and beach), alluvial wadi fill, and evaporitic sabkha deposits.) Holocene |
| 17 | 27.822550427002 | 31.197101549788 | 2.5 | 92.5 | -19.9 | 30.3 | 0.1457142857142857 | 37.0 | 0.9623919587448835 | 0.015043216502046625 | 0.022564824753069937 | Layer 1 | The first surface layer (Unconsolidated carbonate sands (dune and beach), alluvial wadi fill, and evaporitic sabkha deposits.) Holocene |
| 18 | 27.805103912347 | 31.190032562286 | 4.5 | 94.5 | -20.2 | 29.3 | 0.1361904761904762 | 36.2 | 0.9653021470031571 | 0.01387914119873717 | 0.020818711798105752 | Layer 1 | The first surface layer (Unconsolidated carbonate sands (dune and beach), alluvial wadi fill, and evaporitic sabkha deposits.) Holocene |
| 19 | 27.783611829077 | 31.183468502463 | 4.5 | 94.5 | -20.2 | 29.3 | 0.1361904761904762 | 36.2 | 0.9653021470031571 | 0.01387914119873717 | 0.020818711798105752 | Layer 1 | The first surface layer (Unconsolidated carbonate sands (dune and beach), alluvial wadi fill, and evaporitic sabkha deposits.) Holocene |
| 20 | 27.764648226191 | 31.17715690648 | 5.5 | 95.5 | -20.4 | 28.8 | 0.13142857142857142 | 35.8 | 0.9667308014556723 | 0.013307679417731106 | 0.019961519126596657 | Layer 1 | The first surface layer (Unconsolidated carbonate sands (dune and beach), alluvial wadi fill, and evaporitic sabkha deposits.) Holocene |
| 21 | 27.750235887998 | 31.172865021211 | 5.5 | 95.5 | -20.4 | 28.8 | 0.13142857142857142 | 35.8 | 0.9667308014556723 | 0.013307679417731106 | 0.019961519126596657 | Layer 1 | The first surface layer (Unconsolidated carbonate sands (dune and beach), alluvial wadi fill, and evaporitic sabkha deposits.) Holocene |
| 22 | 27.734053613535 | 31.168825599781 | 7.0 | 97.0 | -20.6 | 28.0 | 0.12380952380952381 | 35.2 | 0.9689806659995001 | 0.01240773360019997 | 0.018611600400299955 | Layer 1 | The first surface layer (Unconsolidated carbonate sands (dune and beach), alluvial wadi fill, and evaporitic sabkha deposits.) Holocene |
| 23 | 27.716354250842 | 31.166048497548 | 6.0 | 96.0 | -20.5 | 28.5 | 0.12857142857142856 | 35.6 | 0.9675796566932346 | 0.012968137322706151 | 0.019452205984059223 | Layer 1 | The first surface layer (Unconsolidated carbonate sands (dune and beach), alluvial wadi fill, and evaporitic sabkha deposits.) Holocene |
| 24 | 27.696884951879 | 31.159989365404 | 5.75 | 95.75 | -20.4 | 28.6 | 0.12952380952380954 | 35.7 | 0.9672973957802626 | 0.013081041687894951 | 0.019621562531842426 | Layer 1 | The first surface layer (Unconsolidated carbonate sands (dune and beach), alluvial wadi fill, and evaporitic sabkha deposits.) Holocene |
| 25 | 27.867051681774 | 31.203665609611 | 2.0 | 42.0 | -19.9 | 30.5 | 0.14761904761904762 | 37.2 | 0.9618013425514456 | 0.015279462979421786 | 0.022919194469132678 | Layer 1 | The first surface layer (Unconsolidated carbonate sands (dune and beach), alluvial wadi fill, and evaporitic sabkha deposits.) Holocene |
| 26 | 27.855673520043 | 31.196596622109 | 5.5 | 95.5 | -20.4 | 28.8 | 0.13142857142857142 | 35.8 | 0.9667308014556723 | 0.013307679417731106 | 0.019961519126596657 | Layer 1 | The first surface layer (Unconsolidated carbonate sands (dune and beach), alluvial wadi fill, and evaporitic sabkha deposits.) Holocene |
| 27 | 27.841008333811 | 31.18877024309 | 9.5 | 394.5 | -21.0 | 26.8 | 0.11238095238095239 | 34.2 | 0.9722740968051966 | 0.011090361277921349 | 0.016635541916882022 | Layer 1 | The first surface layer (Unconsolidated carbonate sands (dune and beach), alluvial wadi fill, and evaporitic sabkha deposits.) Holocene |
| 28 | 27.825331755425 | 31.183720966303 | 11.25 | 396.25 | -21.2 | 25.9 | 0.1038095238095238 | 33.5 | 0.9746815890443855 | 0.010127364382245777 | 0.015191046573368661 | Layer 1 | The first surface layer (Unconsolidated carbonate sands (dune and beach), alluvial wadi fill, and evaporitic sabkha deposits.) Holocene |
| 29 | 27.809402329001 | 31.178166761837 | 13.75 | 398.75 | -21.6 | 24.6 | 0.09142857142857144 | 32.5 | 0.9780669637322705 | 0.008773214507091806 | 0.01315982176063771 | Layer 1 | The first surface layer (Unconsolidated carbonate sands (dune and beach), alluvial wadi fill, and evaporitic sabkha deposits.) Holocene |
| 30 | 27.793978598654 | 31.171602702014 | 12.5 | 397.5 | -21.4 | 25.3 | 0.0980952380952381 | 33.0 | 0.9762574321862602 | 0.009497027125495928 | 0.01424554068824389 | Layer 1 | The first surface layer (Unconsolidated carbonate sands (dune and beach), alluvial wadi fill, and evaporitic sabkha deposits.) Holocene |
| 31 | 27.776026387922 | 31.167058352906 | 11.0 | 396.0 | -21.2 | 26.0 | 0.10476190476190476 | 33.6 | 0.9744166959354927 | 0.010233321625802908 | 0.015349982438704361 | Layer 1 | The first surface layer (Unconsolidated carbonate sands (dune and beach), alluvial wadi fill, and evaporitic sabkha deposits.) Holocene |
| 32 | 27.754534304652 | 31.159989365404 | 11.0 | 396.0 | -21.2 | 26.0 | 0.10476190476190476 | 33.6 | 0.9744166959354927 | 0.010233321625802908 | 0.015349982438704361 | Layer 1 | The first surface layer (Unconsolidated carbonate sands (dune and beach), alluvial wadi fill, and evaporitic sabkha deposits.) Holocene |
| 33 | 27.734306461574 | 31.155949943974 | 11.5 | 396.5 | -21.3 | 25.8 | 0.10285714285714287 | 33.4 | 0.9749458359364429 | 0.010021665625422845 | 0.015032498438134263 | Layer 1 | The first surface layer (Unconsolidated carbonate sands (dune and beach), alluvial wadi fill, and evaporitic sabkha deposits.) Holocene |
| 34 | 27.713825770457 | 31.151658058705 | 9.0 | 394.0 | -20.9 | 27.0 | 0.11428571428571428 | 34.4 | 0.9717318733283925 | 0.011307250668643012 | 0.016960876002964517 | Layer 1 | The first surface layer (Unconsolidated carbonate sands (dune and beach), alluvial wadi fill, and evaporitic sabkha deposits.) Holocene |
| 35 | 27.697137799918 | 31.148628492633 | 7.5 | 392.5 | -20.7 | 27.8 | 0.1219047619047619 | 35.0 | 0.9695362964531978 | 0.012185481418720868 | 0.0182782221280813 | Layer 1 | The first surface layer (Unconsolidated carbonate sands (dune and beach), alluvial wadi fill, and evaporitic sabkha deposits.) Holocene |
| 36 | 27.870338706274 | 31.189275170768 | 3.25 | 43.25 | -20.0 | 29.9 | 0.1419047619047619 | 36.7 | 0.9635645707179202 | 0.014574171712831914 | 0.02186125756924787 | Layer 1 | The first surface layer (Unconsolidated carbonate sands (dune and beach), alluvial wadi fill, and evaporitic sabkha deposits.) Holocene |
| 37 | 27.860983328851 | 31.180438936391 | 7.0 | 97.0 | -20.6 | 28.0 | 0.12380952380952381 | 35.2 | 0.9689806659995001 | 0.01240773360019997 | 0.018611600400299955 | Layer 1 | The first surface layer (Unconsolidated carbonate sands (dune and beach), alluvial wadi fill, and evaporitic sabkha deposits.) Holocene |
| 38 | 27.846570990657 | 31.174127340407 | 11.0 | 396.0 | -21.2 | 26.0 | 0.10476190476190476 | 33.6 | 0.9744166959354927 | 0.010233321625802908 | 0.015349982438704361 | Layer 1 | The first surface layer (Unconsolidated carbonate sands (dune and beach), alluvial wadi fill, and evaporitic sabkha deposits.) Holocene |
| 39 | 27.831400108349 | 31.167058352906 | 12.5 | 722.5 | -21.4 | 25.3 | 0.0980952380952381 | 33.0 | 0.9762574321862602 | 0.009497027125495928 | 0.01424554068824389 | Layer 1 | The first surface layer (Unconsolidated carbonate sands (dune and beach), alluvial wadi fill, and evaporitic sabkha deposits.) Holocene |
| 40 | 27.815470681925 | 31.16150414844 | 14.5 | 724.5 | -21.7 | 24.3 | 0.08857142857142858 | 32.2 | 0.9788330562016808 | 0.008466777519327668 | 0.0127001662789915 | Layer 1 | The first surface layer (Unconsolidated carbonate sands (dune and beach), alluvial wadi fill, and evaporitic sabkha deposits.) Holocene |
| 41 | 27.794231446693 | 31.155192552456 | 15.0 | 725.0 | -21.8 | 24.0 | 0.08571428571428572 | 32.0 | 0.9795935556024775 | 0.008162577759008997 | 0.012243866638513496 | Layer 1 | The first surface layer (Unconsolidated carbonate sands (dune and beach), alluvial wadi fill, and evaporitic sabkha deposits.) Holocene |
| 42 | 27.772739363422 | 31.150143275669 | 13.0 | 723.0 | -21.5 | 25.0 | 0.09523809523809523 | 32.8 | 0.9770367356380253 | 0.009185305744789905 | 0.013777958617184855 | Layer 1 | The first surface layer (Unconsolidated carbonate sands (dune and beach), alluvial wadi fill, and evaporitic sabkha deposits.) Holocene |
| 43 | 27.756051392883 | 31.141054577453 | 13.5 | 723.5 | -21.6 | 24.8 | 0.09333333333333334 | 32.6 | 0.9775531078583057 | 0.008978756856677718 | 0.013468135285016574 | Layer 1 | The first surface layer (Unconsolidated carbonate sands (dune and beach), alluvial wadi fill, and evaporitic sabkha deposits.) Holocene |
| 44 | 27.738604878228 | 31.137772547541 | 12.0 | 722.0 | -21.4 | 25.5 | 0.1 | 33.2 | 0.9757347150570881 | 0.009706113977164746 | 0.014559170965747116 | Layer 1 | The first surface layer (Unconsolidated carbonate sands (dune and beach), alluvial wadi fill, and evaporitic sabkha deposits.) Holocene |
| 45 | 27.720399819457 | 31.125906747092 | 13.5 | 723.5 | -21.6 | 24.8 | 0.09333333333333334 | 32.6 | 0.9775531078583057 | 0.008978756856677718 | 0.013468135285016574 | Layer 1 | The first surface layer (Unconsolidated carbonate sands (dune and beach), alluvial wadi fill, and evaporitic sabkha deposits.) Holocene |
| 46 | 27.88095832389 | 31.174379804247 | 3.0 | 43.0 | -20.0 | 30.0 | 0.14285714285714285 | 36.8 | 0.9632724908960749 | 0.014691003641570034 | 0.022036505462355048 | Layer 1 | The first surface layer (Unconsolidated carbonate sands (dune and beach), alluvial wadi fill, and evaporitic sabkha deposits.) Holocene |
| 47 | 27.868063073928 | 31.16529110603 | 8.0 | 98.0 | -20.8 | 27.5 | 0.11904761904761904 | 34.8 | 0.9703646694035128 | 0.011854132238594856 | 0.01778119835789228 | Layer 1 | The first surface layer (Unconsolidated carbonate sands (dune and beach), alluvial wadi fill, and evaporitic sabkha deposits.) Holocene |
| 48 | 27.853145039658 | 31.155949943974 | 11.5 | 396.5 | -21.3 | 25.8 | 0.10285714285714287 | 33.4 | 0.9749458359364429 | 0.010021665625422845 | 0.015032498438134263 | Layer 1 | The first surface layer (Unconsolidated carbonate sands (dune and beach), alluvial wadi fill, and evaporitic sabkha deposits.) Holocene |
| 49 | 27.831905804426 | 31.144336607364 | 12.5 | 722.5 | -21.4 | 25.3 | 0.0980952380952381 | 33.0 | 0.9762574321862602 | 0.009497027125495928 | 0.01424554068824389 | Layer 1 | The first surface layer (Unconsolidated carbonate sands (dune and beach), alluvial wadi fill, and evaporitic sabkha deposits.) Holocene |
| 50 | 27.813953593694 | 31.140044722095 | 2.5 | 1087.5 | -19.9 | 30.3 | 0.1457142857142857 | 37.0 | 0.9623919587448835 | 0.015043216502046625 | 0.022564824753069937 | Layer 1 | The first surface layer (Unconsolidated carbonate sands (dune and beach), alluvial wadi fill, and evaporitic sabkha deposits.) Holocene |

Note: Full dataset has 279 rows. Excerpt shown above. For complete data, refer to source Excel.
